# Regressive research: The pitfalls of post hoc data selection in the study of unconscious mental processes

**DOI:** 10.3758/s13423-016-1170-y

**Published:** 2016-10-17

**Authors:** David R. Shanks

**Affiliations:** Division of Psychology and Language Sciences, University College London, 26 Bedford Way, London, WC1H 0AP England

**Keywords:** Unconscious, Subliminal, Implicit, Regression to the mean, Error

## Abstract

Many studies of unconscious processing involve comparing a performance measure (e.g., some assessment of perception or memory) with an awareness measure (such as a verbal report or a forced-choice response) taken either concurrently or separately. Unconscious processing is inferred when above-chance performance is combined with null awareness. Often, however, aggregate awareness is better than chance, and data analysis therefore employs a form of extreme group analysis focusing post hoc on participants, trials, or items where awareness is absent or at chance. The pitfalls of this analytic approach are described with particular reference to recent research on implicit learning and subliminal perception. Because of regression to the mean, the approach can mislead researchers into erroneous conclusions concerning unconscious influences on behavior. Recommendations are made about future use of post hoc selection in research on unconscious cognition.

Regression to the mean is as inevitable as death and taxes.

—Campbell and Kenny (1999, p. ix)

The study of unconscious or implicit cognition has become a central research topic within experimental psychology. It includes its own specialist journals, such as *Consciousness & Cognition*, has prompted the development of sophisticated instruments, such as the Implicit Association Test (Greenwald, McGhee, & Schwartz, 1998) for measuring unconscious processes, and has been fundamental in the specification and characterization of new neurological syndromes (e.g., blindsight; Weiskrantz, 1986). It has also fostered a range of tailored research methods. This article explains and offers an extensive critique of one such widely used method, based on a form of extreme group analysis (Preacher, Rucker, MacCallum, & Nicewander, 2005), for purportedly demonstrating unconscious effects on behavior. In brief, this article raises a major concern over a method that has been in use for over a century and which continues to flourish today.

In general terms the vast majority of studies of unconscious mental processing involve comparing a performance measure (e.g., some assessment of perception or memory) with an awareness measure (such as a verbal report or a forced-choice response) taken either concurrently or separately. For instance, ten Brinke, Stimson, and Carney (2014) presented participants with videos of actors either telling the truth or lying about a theft. Discrimination was at chance under conscious conditions: participants were unable explicitly (directly) to judge which actors were lying and which were telling the truth. In a performance (implicit, unconscious, indirect) test involving speeded responding, in contrast, participants reacted significantly faster following images of truthful than of deceptive actors. Thus, ten Brinke et al. interpreted this as evidence that deceptive cues from the actors can be detected and “leak” into unconscious but not conscious decisions.

Henceforth, the terms *performance* or *performance measure* will be used to refer to the target behavioral (implicit) measure, often a facilitation in the speed or accuracy of responding (priming). The terms *awareness measure* and *report* will be used to refer to the assessment of conscious (explicit) knowledge. When parallel performance and awareness measures are collected, one pattern that might emerge is for performance to show some above-chance sensitivity to a task variable and for the awareness measure to show null sensitivity. In the experiment just described, the performance test showed sensitivity to the manipulated variable (truth/lie), but the awareness test did not. Although the literature contains hundreds of results fitting this description (such as that of ten Brinke et al., 2014), there are rather fewer that have withstood independent replication attempts (Newell & Shanks, 2014; Shanks & St. John, 1994). Indeed the past half-century of research on unconscious cognition, going back at least as far as the classic debate between Greenspoon (1955) and Dulany (1961), has been characterized by repeated cycles in which new ways of demonstrating unconscious processes have been challenged by later investigations. For example, Vuilleumier, Schwartz, Duhoux, Dolan, and Driver (2005) and Butler and Klein (2009) reported evidence of pure implicit memory using rapid serial visual presentation (RSVP). Line drawings of objects were superimposed (e.g., a red umbrella and a green horse) and presented in an RSVP stream, and participants were instructed to attend to one of the colors. Their subsequent conscious recognition for unattended items was found to be not reliably better than chance, but these objects sustained a repetition priming effect that the authors interpreted as an unconscious process. Extensive efforts by Berry, Shanks, Li, Rains, and Henson (2010) to replicate these findings were unsuccessful, however.

Moreover, when performance shows some above-chance sensitivity at the same time that a report measure shows null sensitivity, there is often the concern that the absence of statistically significant evidence for awareness is (mis)interpreted as evidence of absence (Reingold & Merikle, 1988; Schmidt & Vorberg, 2006). For example, many researchers have reported chance-level awareness in implicit learning studies employing a contextual cuing task (the task is described in more detail later). Vadillo, Konstantinidis, and Shanks (2016) conducted a meta-analysis of 67 experiments, reported with sufficient data to be analyzed. Although all these studies yielded nonsignificant awareness scores and hence concluded that participants lacked awareness of the crucial variable, Vadillo et al. found a significant meta-analytic awareness effect (Cohen’s *d* = 0.16, 95 % CI [0.10, 0.22]) when they were pooled, suggesting that participants’ learning in these experiments was in fact conscious. Vadillo et al. (2016) also found that the average power of these awareness tests was about 0.2. Overreliance on null hypothesis significance testing (NHST) can lead researchers to misinterpret null results from underpowered awareness tests as false negatives.

If there are very few studies of unconscious cognition in which above-chance performance is combined with compelling evidence of chance-level scores on a high-powered awareness test, then what other methodological approaches might provide evidence of unconscious cognition? Some researchers compute performance–report correlations (or the slope of the regression line) on the assumption that if such correlations are close to zero, then it seems implausible that awareness is causally associated with behavioral performance. But once again, absence of evidence is not the same as evidence of absence: A nonsignificant correlation coefficient implies that there is insufficient evidence to reject the null hypothesis (no association); it does not prove that the null hypothesis is true (see Miller, 2000).

As an illustration, Conci and von Mühlenen (2011, Experiment 2) estimated the correlation between the results of an awareness test and a performance (priming) measure and reported *r* = .14, *p* = .62. This lack of significant correlation seems on the face of it to provide support for the claim that learning was unconscious, but without knowing the confidence interval on the correlation coefficient, it is impossible to judge how much weight to place on such a null result. In fact, the 95 % CI on the correlation coefficient has lower and upper limits of -.42 and .62. Thus, the data are compatible with a true correlation in excess of .6. Obviously, this estimation is too imprecise to permit any strong conclusions to be drawn, a problem that applies to many studies that rely on the same strategy. A nonsignificant performance-report correlation would only be theoretically relevant if the confidence interval was narrow (the estimate was precise) and included zero. This, in turn, requires high-powered tests, which are rarely undertaken (Vadillo et al., 2016). Tests of stochastic independence, once popular in research on implicit and explicit memory, fell out of favor for similar reasons (see Poldrack, 1996).

Moreover, correlations or regression slopes close to zero can be generated by models which assume that the performance and report measures are based on a common underlying latent process (Miller, 2000). A more sophisticated approach is to estimate the regression intercept, namely the level of performance when awareness is zero (Greenwald, Klinger, & Schuh, 1995). I return to this method briefly in the Conclusions section.

## Post hoc data selection

As the limitations of the methods described previously have become clear, researchers have turned to other methods, including post hoc data selection, the major focus of this article. Post hoc selection has been in widespread use (as described in a later section) since the earliest days of experimental psychology (e.g., Lazarus & McCleary, 1951; Peirce & Jastrow, 1884; Williams, 1938).

In this procedure, data from participants whose awareness test score is below some cutoff are analyzed separately from the entire group. The cutoff can either be an appropriate baseline score (typically zero on an awareness questionnaire or 50 % on a two-alternative forced-choice test) or could be applied by a test of statistical significance. In the latter case, an appropriate test is applied participant by participant, and data are only included from those whose awareness score is not significantly greater than zero.^1^ For simplicity it is assumed in the following discussion that the former cutoff (zero) is employed. For this subset of *unaware* participants, their mean score on the performance measure is then calculated. If this score is reliably greater than an appropriate baseline for that test, then it is concluded that true unconscious cognition has been demonstrated. In many studies, particularly those assessing subliminal perception (see below), it is items or trials rather than participants to which the awareness cutoff is applied, but the rationale is the same. In these cases, items or trials are selected post hoc for further analysis on the basis that the participant reported no awareness of the stimulus.

The post hoc method, as defined here, must be distinguished from at least two other superficially similar methods for evaluating bivariate *X*–*Y* data. A common analytic technique is to select individuals scoring at both extremes of *Y*—say the top and bottom quartiles—and evaluate the *X*–*Y* relationship across these selected subgroups. Called *extreme groups analysis*, this method has considerable value when data collection is costly. For example, researchers might measure video-game playing across a large sample of individuals by means of a questionnaire, and then administer a time-consuming battery of cognitive ability tests only to those reporting very high or very low hours per week spent playing video games. As well as providing a wider discussion of some of the issues raised by the extreme group approach, Preacher et al. (2005) noted, in passing, another method that they termed *post hoc subgrouping*. This refers to performing extreme groups analysis—examining the *X*–*Y* relationship across subgroups defined by high and low scores on *Y—*even when *X* data have already been collected across the full range of *Y*. The method under consideration here, post hoc data selection, is similar to post hoc subgrouping except that interest is focused on a subgroup at only one extreme of *Y*. Evaluations show that the sacrifice of statistical power that results from dropping data from the middle of the distribution in the post hoc subgrouping method typically has minimal compensating benefits, and accordingly Preacher et al. (2005) were dismissive of the method. But post hoc *data selection* is different in an important way: if the cutoff has a theoretical rationale established a priori (e.g., a zero-point on an awareness measurement scale), then the method may provide a useful tool for investigators wishing to divide a distribution into qualitatively distinct components reflecting conscious and unconscious processing.

The logic of the post hoc data selection method is simple and intuitive, and this likely explains in part its growing usage in the field. When one analyzes group data, there is always the challenge to prove that awareness is at chance. As noted above, NHST is singularly unsuited to providing evidence for a null effect. But if a subgroup of participants is selected precisely on the basis that each of them scores at or below chance on the awareness measure, then it seems unarguable that this subgroup lacks awareness of the relevant feature or variable. And if the subgroup is then shown to perform significantly above chance on the associated performance measure, then it seems equally beyond dispute that true unconscious cognition has been documented. Researchers employing the method presume that there are true individual differences in perception, cognition, and learning such that, under the same conditions, one participant becomes aware of a regularity whereas another one doesn’t. The post hoc selection method identifies the “sweet spot” conjunction between experimental conditions and individual differences such that true unconscious performance is revealed in the selected sample of participants.

A highly cited (>700 citations on Google Scholar, June 2015) example of the use of the method illustrates its intuitive appeal. Clark and Squire (1998) presented amnesic and control participants with an eye-blink conditioning procedure in which a tone (or white noise) conditioned stimulus (CS) preceded an air puff to the eye (unconditioned stimulus, US) and other trials in which the white noise (or a tone) occurred without the US. The behavioral measure was differential conditioned responding to the CS paired with the US in comparison to the CS presented alone. After the conditioning phase, participants answered a questionnaire including 17 questions about the temporal relationships between the CSs and the US. Those scoring ≤12 items correct were classified as unaware of the critical conditioning contingency. Clark and Squire (1998) obtained reliable differential conditioning among the selected subgroup of participants and interpreted this as evidence that eye-blink conditioning can occur unconsciously.

Although never explicitly acknowledged by researchers employing the method, it rests on a fundamental intrinsic assumption. Performance and report measures, like all other measures in psychology, are composed of some underlying true score plus measurement error. When data from a group of participants are collected, then all other things being equal, the measurement errors cancel out and the aggregate mean score approximates the mean true score. The post hoc data selection method assumes that *this same principle applies to the selected subgroup*. As will be seen, a key testable prediction follows from this assumption.

The major aim of this article is to show that the logic behind the post hoc data selection method is flawed and that it does not support the conclusions drawn from it. Indeed, because the assumption above is false, it will be argued that the method *cannot* be used to make inferences about unconscious cognition, or at least not in its uncorrected form. In a nutshell, the problem is that the method must yield above-chance performance scores in participants scoring at or below chance in the awareness test, purely for statistical reasons. The key explanatory phenomenon that undermines the logic of the method is regression to the mean.

In previous work (Berry, Shanks, Speekenbrink, & Henson, 2012; Shanks, 2005; Shanks & Berry, 2012; Shanks & Perruchet, 2002; Shanks, Wilkinson, & Channon, 2003; Smyth & Shanks, 2008; Vadillo et al., 2016), we criticized particular uses of post hoc data selection and proposed that it cannot be employed as a method for revealing evidence of unconscious processing or of dissociations between unconscious and conscious processing. The grounds for those claims were that computational models based on a single underlying variable were able to reproduce the signature pattern obtained from the post hoc selection method, and that it therefore could not follow that demonstrations of above-chance performance in participants selected as scoring at or below chance on an awareness test represent evidence for distinct conscious and unconscious processes. Others (Zeelenberg, Plomp, & Raaijmakers, 2003; Sand & Nilsson, 2016) have also highlighted flawed uses of the method. Here, I develop a much broader and more detailed critique of the method and provide recommendations for future research.

## Post hoc data selection and regression to the mean

**Fig. 1:**
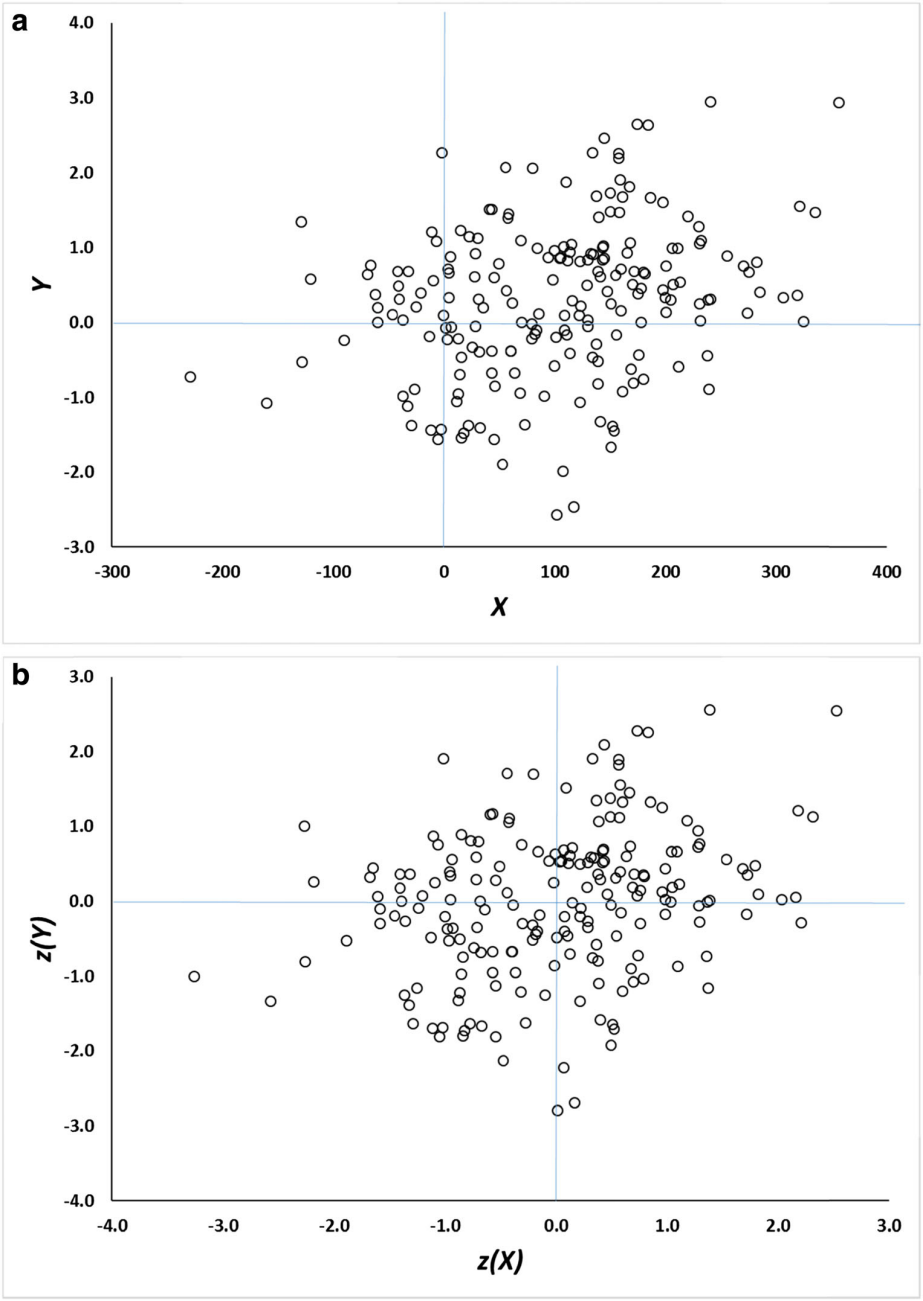
**a** Scatterplot of data generated according to Eqs. 2 and 3. *Y* is assumed to be a measure of awareness, and *X* a measure of performance. **b** The same data as in Panel A, but converted to *z* scores.

As an initial illustration of the problem, consider the data shown in Fig. 1a. This scatterplot represents data from a Monte Carlo simulation in which each point is a hypothetical participant, and the *x-* and *y-*axes represent imaginary performance and awareness measures, respectively, collected for each of 200 participants (throughout this article, awareness is plotted on the *y-*axis and performance on the *x-*axis). The measures are moderately correlated, *r* = 0.28, and each yields a mean score greater than zero, which is taken to be the level of chance or baseline responding.

The *X* values have a mean of approximately $\overline{X}$ = 100, which could reflect a priming score in ms, for example; the *Y* values have a mean of about $\overline{Y}$ = 0.3 which could reflect an explicit recognition *d*′ score, for instance. The exact nature of the measurement scales is not essential to the line of reasoning described below, which is general and also does not depend on where the data points are located in relation to theoretically motivated baseline or chance scores. It also does not depend on the distributional properties of the data (such as whether they are normally distributed), other than the correlation coefficient (Samuels, 1991). The exact model which generated the data will be described later.

In order to carry out a standard post hoc selection analysis on these data, only those simulated participants whose *Y* (awareness) score is less than or equal to zero are retained for further analysis. What is the accompanying mean *X* (performance) score for these participants, $\overline{X}$|*Y* ≤ 0? It has a value of 64, 95 % CI [43, 85], very substantially above zero and not far below the group mean (which in this simulation is 101). This is the essence of post hoc data selection: It reveals that (simulated) unaware participants on average have above-chance performance scores.

**Fig. 2:**
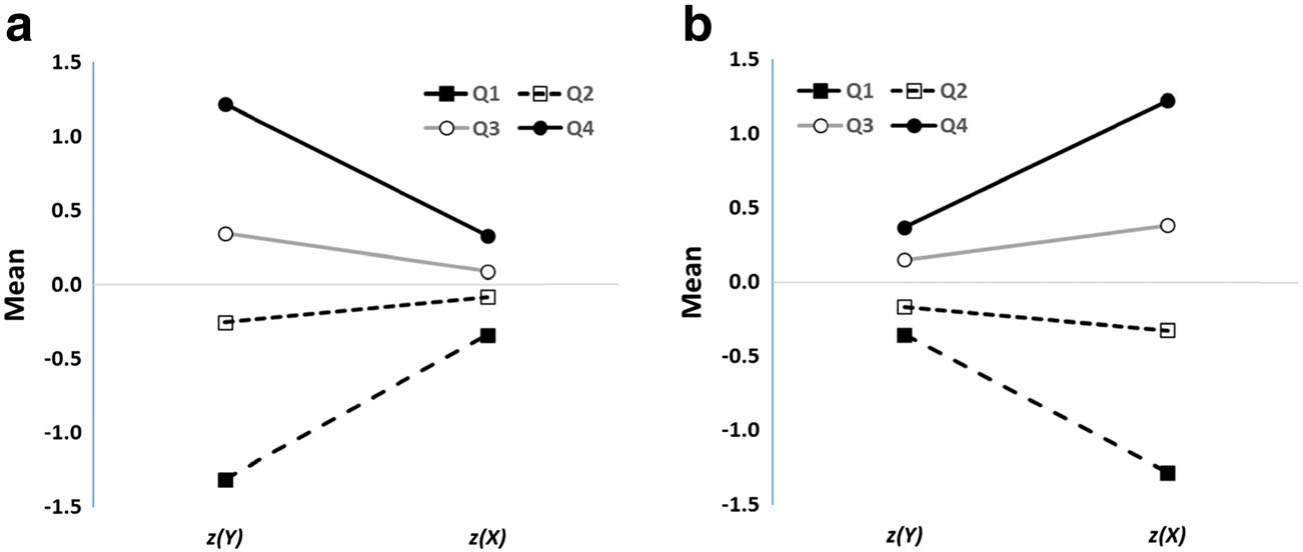
Galton squeeze diagrams for the data in Fig. 1b. **a** mean *z* scores for the bottom (Q1), second (Q2), third (Q3), and top (Q4) quartiles of the *Y* measure plotted against the equivalent *z* scores for *X*. The funnel pattern is regression to the mean. The equivalent analysis based on quartiles for *z(X)* is shown in **b** and indicates the bi-directionality of regression to the mean.

The data in Fig. 1a manifest regression to the mean, but as Campbell and Kenny (1999) noted in their classic monograph on regression artifacts in behavioral research, a scatterplot is a poor method of visualizing this phenomenon. Instead, they built on pioneering work by Francis Galton to devise a much better method for illustrating regression to the mean, the *Galton squeeze diagram*. The first step in constructing such a diagram is to replot the original data in *z*-score space, as in Fig. 1b. This places the *X* and *Y* measures on a common scale and permits them to be compared more directly. Next, the data are partitioned according to their scores on the *z(Y)* dimension. Here this is done by computing quartiles. Finally the mean *z(X)* value for each quartile is calculated and the mean *z(Y)* and *z(X)* for each quartile is plotted in a line graph as in Fig. 2a.

What this shows is that the 25 % of data points from Fig. 1a scoring highest on *Y* have a mean *z(Y)* score of 1.22, but these data points are associated with much less extreme mean *z(X)* score, about 0.33. At the same time, the 25 % of data points scoring lowest on *Y* (mean *z(Y)* = -1.31) are also associated with a much less extreme mean *z(X)* score, about -0.34. This is regression to the mean: Values further away from *z(Y)* = 0 on the left-hand axis regress toward the mean (*z(X)* = 0) on the right-hand axis.

Regression to the mean is an intrinsic property of any bivariate data for which the correlation is less than 1.0 (Campbell & Kenny, 1999). In Fig. 1a, it is reflected by the fact that data points which are extreme on *Y* (either high or low) tend to be less extreme on *X*, and vice versa.^2^ When the variables have the same mean and standard deviation (i.e., are transformed into *z* scores), the magnitude of regression to the mean is constrained by only one factor, namely, the correlation coefficient. The predicted *z(X)* score for a data point measuring *z(Y)* is (see Campbell & Kenny, 1999; Cohen, Cohen, West, & Aiken, 2003, ch. 2)^3^:

1 $$ z(X)=r\kern0.5em z(Y). $$

Thus, if the mean *z(Y)* of the bottom quartile of data points (-1.3) and the correlation coefficient (0.28) are known, the associated mean *z(X)* score can be predicted, in this case -0.36. This is close to the value in Fig. 2a and only differs as a consequence of sampling error. The smaller the correlation, the greater the extent of regression to the mean. In the extreme case where *r* = 0, the expected value of *z(X)* is zero—complete regression to the mean.

In formal terms:

$$ \left|\mathrm{mean}\kern0.5em z\Big(Y\right|Y\le c\left)\kern0.2em \hbox{-} \kern0.2em \mathrm{mean}\kern0.5em z(Y)\left|>\right|\mathrm{mean}\kern0.5em z\right(X\left|Y\le c\right)\kern0.2em \hbox{-} \kern0.2em \mathrm{mean}\kern0.5em z(X)\Big|, $$

where *c* is the relevant cutoff on *Y*. Because mean *z(Y)* = mean *z(X)* = 0, it follows that

$$ \left|\mathrm{mean}\kern0.5em z\Big(Y\right|Y\le c\left)\left|>\right|\mathrm{mean}\kern0.5em z\right(X\left|Y\le c\right)\Big|. $$

Hence, “it is a mathematical necessity that whenever two variables correlate less than perfectly, cases that are at one extreme on one of the variables will, on the average, be less extreme on the other” (Cohen et al., 2003, p. 36).

It is important not to be misled by the funnel shape of Fig. 2a. The data points at the right are as dispersed as those on the left (after all, the scatter of the points is what’s illustrated in the *z*-score graph in Fig. 1b). It’s simply that the expected value of an extreme *Y* score regresses towards the *X* mean. Moreover, a key feature of regression to the mean is that it is bidirectional. Just as short fathers tend to have taller sons, by the same token, short sons tend to have taller fathers. Thus Fig. 2b shows that an exactly analogous funnel pattern arises if it is the *z(X)* rather than *z(Y)* scores that are the basis for the segregation into quartiles: the 25 % of data points scoring lowest on the *z(X)* dimension have a mean of -1.28 on that dimension but a mean of only -0.35 on the *z(Y)* dimension.

This latter illustration has a significant implication. Researchers employing the post hoc method, and who believe that performance can in some circumstances be more sensitive than awareness (see Merikle & Reingold, 1991), might be tempted to predict that it will be easier to select participants post hoc who score above chance on the performance measure and at (or below) chance on the awareness measure than the converse. Yet the previous analysis shows that such a pattern is unlikely to be observed. Just as participants performing below a cutoff on the awareness measure *Y* must have a mean performance *X* score closer (in standard scores) to the group average on *X*, so participants performing below a cutoff on the performance measure *X* must have a mean *Y* awareness score closer to the group average on *Y*.

What are the implications of these demonstrations of regression to the mean for the post hoc data selection method? The answer is simple: When two variables are imperfectly correlated, then regardless of the underlying relationship between the latent variables they measure, it is a statistical certainty that applying an extreme cutoff on one dimension (such as a measure of awareness) will yield a less extreme cutoff for the expected value of the other variable (such as a measure of performance). If the bottom quartile of data points on *Y* are selected, and it is assumed that the subgroup this creates comprises unaware participants, then it is a statistical inevitability that the subgroup’s expected mean score on *X*, $\overline{X}$|*Y* ≤ *c*, will be closer (in standard scores) to the overall mean of *X*, $\overline{X}$, than their mean awareness $\overline{Y}$|*Y* ≤ *c* is to the overall mean awareness score $\overline{\;Y}$. As Eq. 1 shows, it literally could not be otherwise, so long as *r* < 1.0 (Campbell & Kenny, 1999). The only circumstances in which no regression occurs are when *r* = 1.0, but this requires no measurement error, perfectly reliable measures, and a perfect correlation between the latent variables measured by *X* and *Y*.

This analysis assumes that *X* and *Y* are on a common scale, derived via a simple *z* transformation. Estimating regression to the mean with raw scores can raise additional issues (Kenny, 2005), but because the transformation is reversible whatever is true for *z*-transformed data is also true for raw scores. The two analyses of published data presented later in this article are conducted principally on raw scores and hence illustrate that the issues highlighted here apply to the types of measures typically collected in studies of unconscious cognition, even when untransformed.

To illustrate regression in operation, Clark and Squire (1998) employed post hoc selection to obtain evidence of unaware eye-blink conditioning, as highlighted previously. Regression to the mean is sufficient, in principle, to account for this finding without any assumption having to be made about unconscious mental processes. Equation 1 establishes that participants who were selected as being extreme on one measure (awareness) must be less extreme on the other. If the group mean conditioning score ($\overline{X}$) is greater than zero, then the score for participants who were selected on the basis of extreme scores on *Y* will regress toward this mean conditioning score.

Indeed, other aspects of Clark and Squire’s results provide further support for this account. The key conditioning-without-awareness pattern was obtained in only some of the conditions they tested. Specifically, participants selected post hoc as unaware showed robust delay eye-blink conditioning (in delay conditioning the CS and US overlap temporally), but this pattern was not observed when a trace conditioning procedure (in which the CS and US do not overlap) was used. In the latter condition, aware but not unaware participants showed conditioning. Looking in detail at the conditioning–awareness correlation in the different groups, this pattern follows naturally. In delay conditioning, the conditioning–awareness correlation was negligible (*r* ≈ 0), and hence from Eq. 1, substantial regression is predicted and “unaware” participants should show conditioning. In trace conditioning, in contrast, the conditioning–awareness correlation was substantial (*r* ≈ 0.7), and hence little regression is predicted. Consistent with the regression to the mean formula (Eq. 1), evidence of conditioning in participants who were selected post hoc as being unaware was obtained precisely in those conditions where the *X*–*Y* correlation was smallest. Admittedly, the regression account does not explain why the conditioning–awareness correlations were so different in the two cases, but then neither does Clark and Squire’s theory, which is circular: The correlation is low for delay conditioning because learning was unconscious; but the post hoc method, in conjunction with the low correlation, was guaranteed to identify apparently unconscious learning in delay conditioning.

An extreme way of highlighting the pitfalls of the post hoc selection method is to consider the conclusions it would point to when a measure of awareness is completely unreliable. Imagine an awareness test that is equivalent to a coin toss. The selection method would segregate participants into those identified as “aware” and “unaware” by this test, and because the *X*–*Y* correlation would necessarily be zero, there would be complete regression to the mean on *X*. Hence, “unaware” participants would appear to show significant performance (*X*), but this, of course, would be a spurious conclusion.

This section has articulated the major point of this article. In the remaining sections, the regression artifact is further unpacked, generalized to a range of other situations (e.g., binary awareness measures), applied and tested on some real rather than simulated data sets, and placed in historical context.

## Why does regression to the mean occur?

As noted above, the post hoc selection method assumes that the measured variables are composed of some underlying true score plus error, and that when data from a selected subgroup of participants are aggregated, the errors cancel out, and the ensuing mean score approximates the mean true score. To see why this assumption is false, it is necessary to describe the model that generated the data in Fig. 1a. The model begins with a random, normally distributed variable *S* with mean and standard deviation (*σ*_*S*_) equal to 1, and with *S* = 0 representing the baseline of no knowledge. This common underlying variable forms the basis of both the performance (*X*) and report (*Y*) measures. Specifically:

2 $$ X=100\kern0.5em S+30\kern0.5em {e}_X, $$

3 $$ Y=0.30\kern0.5em S+{e}_Y. $$

**Fig. 3:**
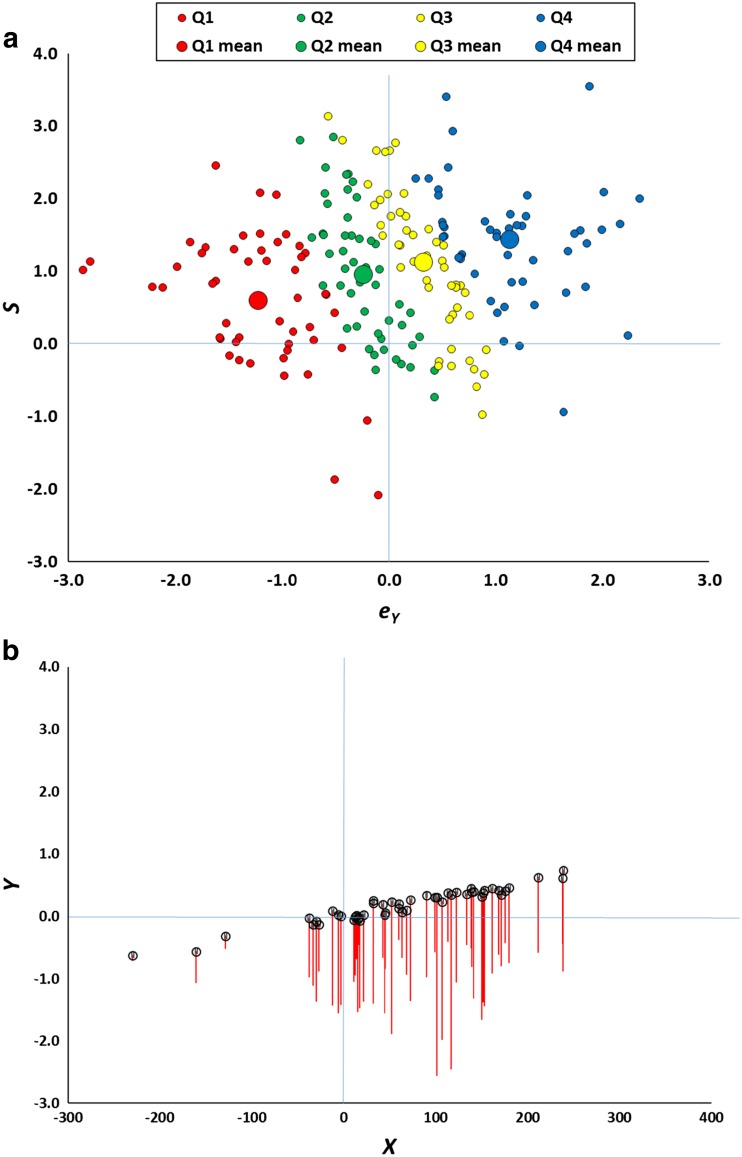
**a** Basis of the regression effect illustrated in Fig. 2. The scatterplot represents the values of *S* and *e*_*Y*_ from Eq. 3 for the data points in Fig. 1a, for the bottom (Q1), second (Q2), third (Q3), and top (Q4) quartiles of *Y*. Large symbols are the means for each quartile. Although the overall mean value of *e*_*Y*_ is zero, for the data points in Q1, the values of *e*_*Y*_ are systematically less than zero, while for those in Q4 they are systematically greater than zero. **b** The figure reproduces all the bottom quartile *Y* data points from Fig. 1a, but decomposes them into their true score and measurement error. The circles mark where each point would lie if *e*_*Y*_ is set to zero. The red line extends to the observed *Y* when *e*_*Y*_ takes its true value. Thus the tip of each red line lies at exactly the same value as depicted in Fig. 1a. (Color figure online)

The variable *S* is first scaled by a factor of 100 in Eq. 1 and combined with normally distributed random error *e*_*X*_, which has a mean of zero and *σ* = 1, to yield that participant’s performance score. This very same value of *S* is scaled by a factor of 0.3 in Eq. 2 and combined with independent error *e*_*Y*_ (again, with mean zero and *σ* = 1) to yield that participant’s awareness score. It is therefore possible to ask whether or not the assumption on which the post hoc selection method rests is correct. Of course, for real behavioral data, this decomposition into true and error components cannot be achieved, but because the data in Fig. 1a were generated from a known model (Eqs. 2 and 3), such a decomposition can be done for these simulated data (for a related simulation, see León & Suero, 2000). That is, we can ask whether the *Y* scores for the bottom quartile have a mean *e*_*Y*_ of (approximately) zero. Figure 3a depicts the original 200 data points, but this time plotting the true value of *S* against *e*_*Y*_ for each point. Overall the mean *e*_*Y*_ is close to zero and the mean value of *S* is close to 1.0, as expected from the model specification.

Contrary to the assumption that the measurement errors have a mean of zero in the selected subgroup, the points representing each quartile are not at all superimposed. Instead, the points comprising the lowest *Y* quartile have *e*_*Y*_ values consistently less than zero, whereas the points comprising the highest quartile have *e*_*Y*_ values consistently greater than zero. The large markers illustrate the mean for each quartile and fall on a line sloping upwards to the right. Although it is the case that points falling in the top quartile have larger true *S* scores than those in the bottom quartile (the mean for the former is higher than the mean for the latter), it is also the case that the quartiles differ considerably in their mean *e*_*Y*_ values. This arises because if a randomly chosen value of *e*_*Y*_ happens to be large and negative, it is much more likely that the resulting *Y* score from Eq. 3 (in which *e*_*Y*_ is one of the components) will be negative than it would be if the value of *e*_*Y*_ were large and positive. The converse is the case if a randomly chosen value of *e*_*Y*_ happens to be large and positive. Put differently, although *e*_*Y*_ and *S* are necessarily uncorrelated, the same is not true of *e*_*Y*_ and (0.30 *S* + *e*_*Y*_), which must be correlated because they incorporate a common term. This correlation means that high positive values of *e*_*Y*_ will be associated with high positive values of the measured variable *Y* (= 0.30 *S* + *e*_*Y*_), and similarly for large negative values. When *e*_*Y*_ is close to zero, *Y* will tend to be too. Krause (2009) uses the term *captures* to describe participants who—because of an extreme amount of measurement error—are falsely selected for inclusion in the extreme group.

The net effect is that the *Y* scores of participants selected post hoc are systematically biased by error components which on average are not zero. On average, the low awareness score of a participant selected by the post hoc method is made up of a “true” underlying score that is not particularly extreme (the mean *S* values for the quartiles are not widely dispersed) and an error component that is extreme (the mean *e*_*Y*_ values for the quartiles are widely dispersed). When the true score *S* is then combined with the error term *e*_*X*_ (which will, on average, have a mean of zero) in Eq. 3, regression to the mean must follow in the resulting expected *X* score. Because *e*_*X*_ and *e*_*Y*_ are uncorrelated, when an observation has an extreme value of *e*_*Y*_, it is very unlikely that *e*_*X*_ will be equally extreme. Once again, I stress that this is not an empirical speculation, but a statistical inevitability. So long as measurement error is greater than zero, the “true” expected level of awareness of a selected participant must, on average, be greater than his or her measured score. That it is error in *Y* scores (*e*_*Y*_) that is crucial is confirmed by the fact that the key regression patterns in Figs. 2 and 3 persist if *e*_*X*_ is fixed at zero, but are abolished if *e*_*Y*_ is zero.

Figure 3b depicts the biasing consequences of the post hoc method in a particularly vivid manner. The figure reproduces all the bottom quartile *Y* data points from Fig. 1a, but decomposes each into its true score and measurement error. The unmarked tips of each red line lie at exactly the same values as depicted in Fig. 1a (hence, if this figure were overlain on Fig. 1a, the tip of each red line would fall exactly on top of a data point in the figure). The circles mark where each point would lie if there were no measurement error associated with the data point, calculated by setting *e*_*Y*_ to zero. The red line therefore extends to the observed *Y* when *e*_*Y*_ takes its true value, and the lengths of the red lines represent exactly the same set of *e*_*Y*_ values graphed for the equivalent (red) data points in Fig. 3a. As Fig. 3b illustrates, the measurement errors are far from random. Instead, they systematically transform data values for which true *Y* > 0 into ones for which observed *Y* < 0. Paradoxically, most of a sample of simulated participants, who all appear to lack any awareness whatsoever, actually have above-chance awareness. The true values of *Y* show an upward slope as *X* increases, and this pattern manifests itself because large values of *X* usually arise from large values of *S*, which in turn yield large values of *Y*.

It bears emphasizing that the model cannot predict above-chance performance in the true absence of awareness. To see this, note that mean awareness $\overline{Y}$ in Eq. 3 can only be at chance (zero) when $\overline{S}$ = 0. Because *S* is identical in Eqs. 2 and 3, then if $\overline{S}$ = 0, $\overline{X}$ = 0 as well, and performance is predicted to be at chance. This confirms that the key qualitative pattern that emerges when data are selected post hoc cannot be proof of true unconscious mental processes. In other words, this is a demonstration that the canonical data pattern that the post hoc method interprets as evidence of unconscious processes can arise from a model in which no such processes exist.

When a researcher collects data from a group of participants, it is entirely reasonable (and indeed correct) to assume that random measurement errors across participants will cancel each other out and that the aggregate measure for the group approximates the true score. It also seems reasonable, but is in fact fallacious, to assume that the same applies to a selected subgroup. The very fact of selecting participants on a nonrandom basis immediately introduces bias in the errors for that subgroup, as participants with extreme error values will be overrepresented. This is impossible to demonstrate in real behavioral data where the generating model is unknown, but is evident when the model is known, as in the simulation described here.

The model analyzed above demonstrates that when dispersion in *X–Y* data is caused solely by measurement error (*X* and *Y* would be perfectly correlated if *e*_*X*_ and *e*_*Y*_ were zero), then we can conceptually explain the ensuing regression to the mean of *Y* on *X* via bias in the values of *e*_*Y*_ among the data points selected post hoc. Measurement error is only one potential cause of regression to the mean: Anything that contributes to an imperfect correlation induces it (Campbell & Kenny, 1999, p. 30). Can the model be generalized to accommodate cases where *X–Y* dispersion is caused at least in part by other, nonrandom factors? Imagine that participant *i*’s scores on *X* and *Y* are attributable partly to a common variable *S* ^*i*^_*C*_, but also partly to factors (*S* ^*i*^_*X*_ and *S* ^*i*^_*Y*_) that are unique to the performance and report tests, respectively:

4 $$ X={S^i}_C+{S^i}_X+{e}_X, $$

5 $$ Y={S^i}_C+{S^i}_Y+{e}_Y. $$

Each term could be weighted differentially (as in Eqs. 2 and 3), but these weights are omitted here for simplicity. This is a more realistic model of report and performance which assumes a factor common to both types of test as well as unique factors. For instance, general attentiveness (*S* ^*i*^_*C*_) might vary across participants in such a way that highly attentive individuals tend to score high on both types of test. At the same time, “intuitive” individuals might score high on unconscious processing (*S* ^*i*^_*X*_) independently of conscious (*S* ^*i*^_*Y*_) processing, and “deliberative” individuals might score high on conscious processing independently of unconscious processing. The simpler model (Eqs. 2 and 3) is of course a special case of the more complex model specified by Eqs. 4 and 5.

In this model, *S* ^*i*^_*X*_ and *S* ^*i*^_*Y*_ are independent, but constant within participants and will therefore tend to weaken the correlation between *X* and *Y*. With the error terms *e*_*X*_ and *e*_*Y*_ further weakening that correlation, the model encapsulated in Eqs. 4 and 5 thus predicts robust regression to the mean, of magnitude determined by Eq. 1. In the case where *e*_*X*_ and *e*_*Y*_ are zero, the ensuing regression would not, of course, be attributable to the phenomenon illustrated in Fig. 3 (bias in *e*_*Y*_), but to a related effect: The independent distribution of *S* ^*i*^_*X*_ and *S* ^*i*^_*Y*_ means a below-chance-level score on *Y* selected post hoc would be likely to incorporate a negative value of *S* ^*i*^_*Y*_. For such a score, it is bias in *S* ^*i*^_*Y*_ rather than in *e*_*Y*_ that would create regression to the mean, and a result similar to that depicted in Fig. 3 would ensue, with *S* ^*i*^_*Y*_ replacing *e*_*Y*_. Although situations where *e*_*X*_ and *e*_*Y*_ are zero are unlikely to have any meaning within behavioral research, this example illustrates that regression to the mean would still be a statistical inevitability even if they were.

**Fig. 4:**
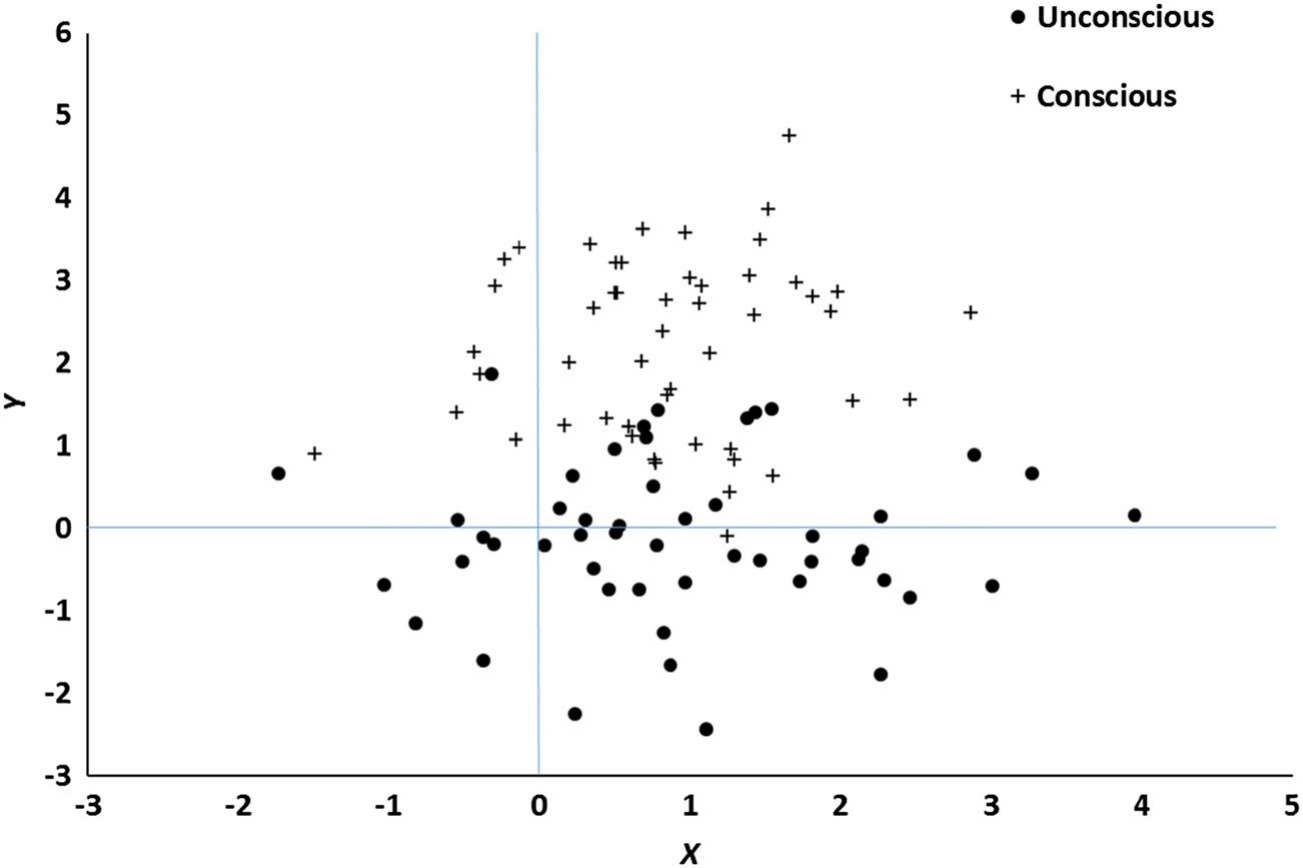
Scatterplot of data generated according to the mixture model described in the text. *Y* is assumed to be a measure of awareness and *X* a measure of performance. Crosses represent simulated participants whose performance is truly conscious and circles ones whose performance is truly unconscious.

A further model variant is one in which there are genuinely two distinct subpopulations of participants, one in which awareness and performance are correlated and another in which they are uncorrelated. Such a mixture model allows us to ask whether the post hoc selection method has diagnostic value when at least some participants truly do show an unconscious performance effect. For instance, imagine that the performance of some participants (True Aware) is determined by a model similar to that of Eqs. 2 and 3, such that *X* = *S* + *e*_*X*_ and *Y* = 2 + *S* + *e*_*Y*_, where *e*_*X*_, *e*_*Y*_ ~ *N*(0,1) and *S* ~ *N*(1,1). In contrast, the performance of other participants (True Unaware) is determined by a model in which *X* = *S* + *e*_*X*_ and *Y* = *e*_*Y*_. Conscious (*Y*) and unconscious (*X*) measures will tend to be correlated in True Aware participants because each depends on the common variable *S*. But *Y* and *X* will be uncorrelated in True Unaware participants, for whom *Y* is simply a random value drawn from a distribution with mean zero. Figure 4 shows a resulting scatterplot for 200 simulated participants, 100 from each subgroup. Clearly, the True Unaware participants have on average no awareness ($\overline{Y}$ = 0), but above-chance performance ($\overline{X}$ > 0).

If the post hoc selection method is applied to these data, with a cutoff at *Y* = 0, we obtain, naturally, evidence of unconscious processing. In this case this would be a correct inference because the data are derived from participants in whom true latent knowledge *S* influences performance, but not awareness. However, unless the distribution of *Y* scores shows evidence of bimodality, the method can provide no proof that the data come from such a mixture model. As the examples described previously show, the same qualitative pattern ($\overline{X}$ > 0 when $\overline{Y}$ = 0) can arise even when the underlying model does not permit a distinction between conscious and unconscious processes at the level of the latent processes. So long as regression to the mean occurs, the pattern is inevitable. And the only condition required for regression to the mean is that *X* and *Y* are imperfectly correlated.

To summarize, in this section I have shown that regression to the mean is a statistical inevitability whenever *X* and *Y* are imperfectly correlated, and the magnitude of regression (in *z* space) is determined solely by *r*. The reason it occurs is that whatever is the source of between-participants variation (be it measurement error or a nonrandom factor or both) will be unevenly distributed across a subsample formed on the basis of *Y*. As soon as one collects a sample post hoc on the basis of a cutoff on the *Y* variable, members of that sample will tend to have biased values for the factor underlying that variation, because members for whom the values are extreme are more likely to meet the cutoff criterion. The bias will disappear in the *X* measure due to regression to the mean. Even if the sample includes truly unaware participants, the post hoc method provides no mechanism for proving as much.

## Regression to the mean for binary measures

The analysis provided above assumes continuous measures of conscious and unconscious processing. However, awareness is frequently measured in a binary way. Many studies on subliminal perception, for example, present participants on each trial with a masked prime stimulus followed by a target stimulus and measure responding to the target. Such studies determine whether the prime exerts any influence on the target response, compared to an unprimed baseline. At the end of each trial the participant reports whether the prime was seen (conscious) or unseen (unconscious). The investigator then performs post hoc data selection at the level of items rather than participants. That is to say, all items for which participants made “unseen” reports are aggregated and target responding is assessed for this subset of items.

In this section I show that this method is undermined by regression to the mean in an analogous way to that described previously (for other criticisms of this technique, see Schmidt, 2015). To demonstrate this I once again construct a simple model. In this case, the latent variable *S* is a random Bernoulli variable akin to a coin toss and with values zero or one, each occurring with probability 0.5. *S* can be thought of as the underlying true binary state of the participant when a given prime stimulus is presented. Next, it is assumed that the behavioral response *X*, which might, for example, be a dependent measure in milliseconds, is determined by

6 $$ X=100\kern0.5em S+30\kern0.5em {e}_X, $$

where *e*_*X*_ ~ *N*(0,1). From this it follows that *X* will have an overall mean of zero (there will be no priming) when the participant is in the true “unseen” state (*S* = 0), but will have a mean of 100 (substantial priming) when she is in the true “seen” state (*S* = 1).

The model assumes that the participant’s binary verbal report (“seen”/”unseen”) depends on the same latent state variable *S*:

7 $$ Y=\left\{\begin{array}{lll}1\hfill & \mathrm{if}\hfill & S+{w}_Y{e}_Y\ge 0.5\hfill \\ {}0\hfill & \mathrm{if}\hfill & S+{w}_Y{e}_Y<0.5,\hfill \end{array}\right. $$

where *e*_*Y*_ ~ *N*(0,1) and *w*_*Y*_ is a weight. Consider the case first of all where *w*_*Y*_ is zero. Under these conditions a simple correspondence exists such that *Y* = *S*: measured awareness is perfectly aligned with the underlying true state. Next, consider the alternative case where *w*_*Y*_ = 1. If *S* = 0 and the amount of error *e*_*Y*_ is close to zero, *Y* will also be zero. But on those trials when by chance *e*_*Y*_ is greater than or equal to 0.5, *Y* will cross the 0.5 threshold and switch from zero to one. Conversely, if *S* = 1 and *e*_*Y*_ is close to zero, *Y* will also be 1, but on those trials where *e*_*Y*_ is less than -0.5, *Y* will cross the 0.5 threshold and switch from one to zero. The error term *e*_*Y*_ introduces trials where the measured and true states *Y* and *S* diverge. The weight *w*_*Y*_ serves to modulate the impact of error: the larger *w*_*Y*_, the more error trials there will be. As before, this model cannot predict (subliminal) performance in the true absence of awareness ($\overline{S}$ = 0). If $\overline{S}$ = 0, then $\overline{X}$ = 0 too.

**Fig. 5:**
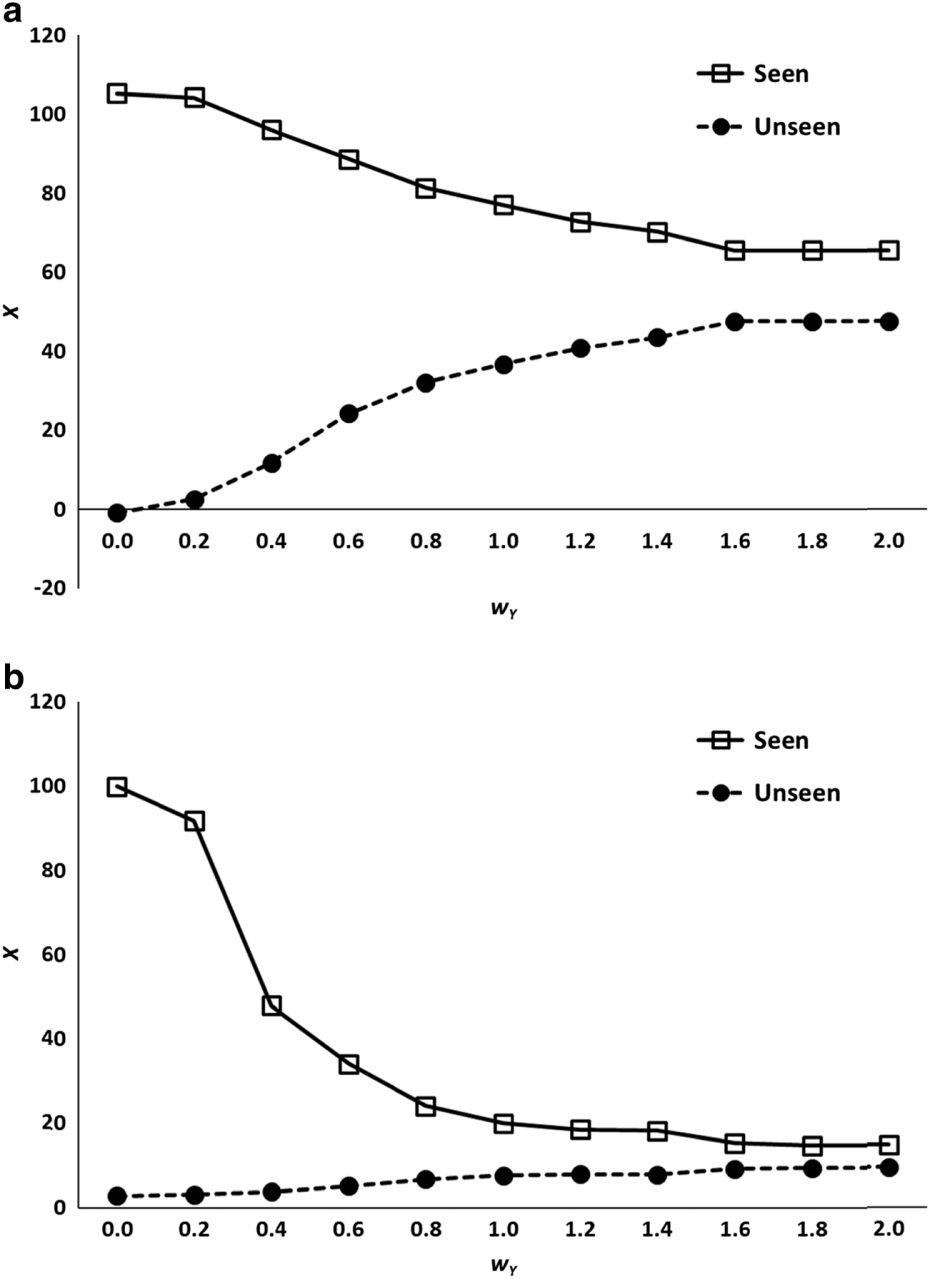
**a** Simulated results from the binary awareness model described in Eqs. 6 and 7. *Y* is assumed to be a measure of awareness, and *X* a measure of performance. Squares represent trials in which participants report conscious experience of the stimulus (Seen), and circles represent trials in which they report no conscious experience (Unseen). **b** Predictions of the model when the probability of *S* = 1 is set to 0.1 rather than 0.5.

Figure 5a presents results from this model across 200 simulated participants, plotting the mean level of priming ($\overline{X}$) obtained for different values of *w*_*Y*_ when post hoc selection is applied. In the complete absence of error on *Y*, no priming is obtained on “unseen” trials, while substantial priming (=100) occurs on “seen” trials. As *w*_*Y*_ increases, so does the amount of priming on unseen trials. Thus, simply as a consequence of measurement error, the model generates an apparent but in fact wholly artifactual subliminal priming effect. When the contribution of error on *Y* becomes quite large, the degree of priming on seen and unseen trials tends towards convergence.

In this example seen and unseen trials are assumed to be equally frequent, but this may be an unrealistic assumption. In many subliminal perception experiments the frequency of seen trials is much lower than 50 %, presumably because the stimuli are genuinely below or at least close to the awareness threshold. In the experiments on brightness discrimination by Harris, Schwarzkopf, Song, Bahrami, and Rees (2011), for instance, the proportion of seen trials was only 12 %–26 % in different conditions. Although this alters the precise quantitative behavior of the model described in Eqs. 6 and 7, it does not alter its qualitative predictions. Figure 5b shows the model’s output when the probability of *S* having the value 1 is reduced from 50 % to 10 %. When *w*_*Y*_ = 0.6, for example, the percentage of seen trials is 22 % (this is larger than 10 % because many trials with *S* = 0 become seen trials as a result of the error component of Eq. 7). Although the expected mean level of priming (*X*) obtained for different values of *w*_*Y*_ is lower than in Fig. 5a, the level again is greater than zero and increases with *w*_*Y*_, yielding substantial priming on unseen trials.

Regression to the mean is sufficient to induce apparent unconscious priming in a model that does not permit true unconscious effects at the level of the underlying latent psychological state. It does so because of the asymmetry of “captures,” cases where as a result of measurement error true states with *S* = 1 become unseen reports (*Y* = 0). Such cases regress towards the mean on *X*, yielding substantial priming scores. Only in the unrealistic case where measurement error is completely absent does this effect attenuate completely.

## Testing a key prediction of the regression account

If the low awareness scores of participants selected post hoc in some sense reflect their true awareness, as researchers employing the method assume, then a simple prediction can be derived. Suppose such participants are tested a second time on the awareness test. On this account their average awareness scores should be similar to those in the first test. Their low scores on Test 1 are assumed to reflect some true underlying (non)awareness, plus random measurement error. On Test 2 the underlying awareness is the same, but combined with new and uncorrelated measurement error. Because the latter is unbiased (has a mean of zero), the mean awareness of a group of participants selected by this method should be the same on Tests 1 and 2.

The regression account makes a strongly contrasting prediction, namely that on Test 2 these participants will score higher than on Test 1. Because the post hoc selection method collects extreme data on the awareness measure, biased by extreme *e*_*Y*_ error components, and because scores on two awareness subtests will invariably be imperfectly correlated, scores on Test 1 will regress to the mean on Test 2 (and vice versa). Thus, the regression account makes a testable prediction which is strongly at variance with an assumption of the post hoc selection method.

To test this novel prediction, I reanalyzed data from a contextual cuing study by Smyth and Shanks (2008, Experiment 1). This experiment replicated and extended an earlier one by Chun and Jiang (2003) that had employed post hoc data selection among its analyses. Full details of the procedure are reported in the original article and in the main are not crucial here, but in brief the experiment comprised the following elements. Forty participants completed 24 blocks of a contextual cuing experiment, each comprising 24 visual search trials in which participants located a target (the letter *T* inverted by 90°) among 11 distractors (*L*s). On locating the target, they pressed one of two response keys as fast as possible to indicate its orientation. The key manipulation is that some of the patterns of distractors repeated during the experiment (once per block) and for these patterns the target was always in the same location (though its orientation was unpredictable). Contextual cuing experiments ask whether participants can learn about the predictiveness of repeating configurations of objects, as indicated by faster RTs to repeating compared to random (nonrepeating) displays. The RT difference for nonrepeating and repeating patterns was calculated for each participant across the final six blocks of the experiment, and this is taken as each individual’s performance score in the following discussion. Contextual cuing is a reliable phenomenon, and 30/40 (75 %) of the participants showed a numerical learning effect, *M* = 80.6 ms, 95 % CI [37.9, 123.2].

At the end of the contextual cuing stage, participants’ awareness was assessed via a generation test in which on each trial a display was presented, in which the target *T* had been replaced with another distractor *L* and the participant was required (under no time pressure) to indicate which quadrant of the screen contained this “hidden” target. The test therefore asks participants to make nonverbal reports about the key experimental variable.

**Fig. 6:**
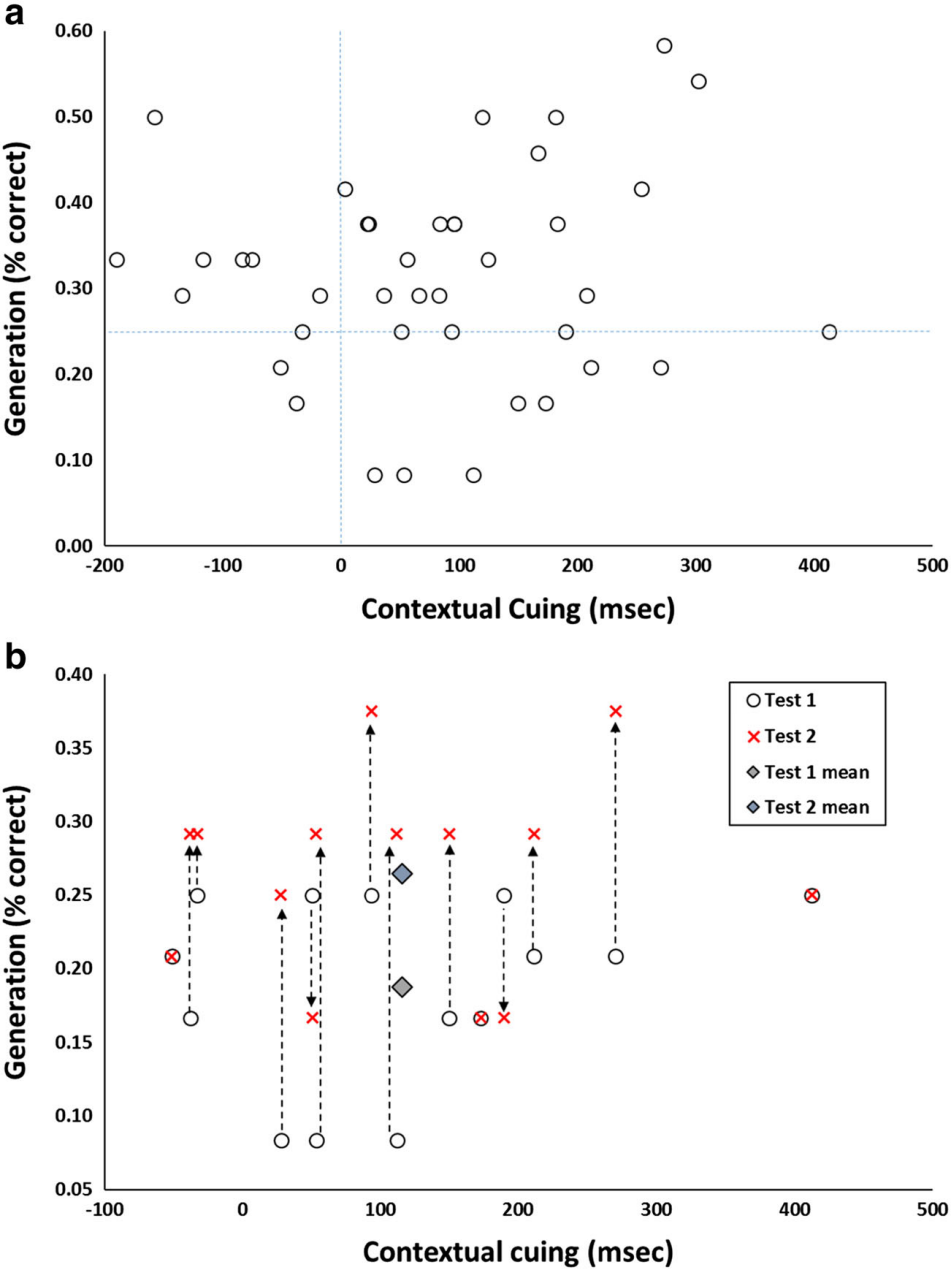
**a** Data from Smyth and Shanks (2008, Experiment 1). Each point represents a participant, plotting the magnitude of contextual cuing (*X*, the mean priming effect in ms across the final six blocks of the experiment) against awareness (*Y*, generation score, percentage correct in Test 1). **b** Data are shown for the 14 participants in Panel A who scored at or below chance (25 %). The open circles reproduce the data from Panel A, and the red crosses show each participant’s score on Test 2. Diamond symbols show the Test 1 and Test 2 means. The majority of generation scores move upwards (become larger) from Test 1 to Test 2, reflecting regression to the mean, and are no longer at or below chance. (Color figure online)

The generation test comprised four blocks of trials each comprising the 12 repeated configurations from the learning stage and 12 random configurations. The latter are ignored in the following analysis, which focuses on performance on the repeated configurations. If contextual cuing yields pure unconscious knowledge then participants should be unable to report the quadrant containing the hidden target (Chun & Jiang, 2003). Because there are four quadrants, chance-level proportion correct is .25. Each participant’s score combined across Blocks 1 and 2 of the generation test (Test 1) and across Blocks 3 and 4 (Test 2) was calculated. Figure 6a is a scatterplot with the performance score (contextual cuing) on the *x-*axis and Test 1 awareness on the *y-*axis.

The group’s performance on Test 1 was .31, 95 % CI [.28, .35]. Consistent with the point made in the introduction, this level is significantly above chance. Group-level assessments of awareness in implicit learning experiments rarely yield scores truly at chance (Vadillo et al., 2016). Thus, post hoc data selection may yield evidence for true implicit learning, even though group performance does not. Figure 6a indicates that 14/40 participants scored at or below chance on the awareness test (Test 1) and hence are selected for further analysis. These data are reproduced in Fig. 6b (note that the axes have been adjusted to make visualization easier). These 14 participants show a strong performance effect, *M* = 115.9 ms, 95 % CI [40.7, 191.2], if anything, slightly greater than the mean for the entire group. Thus, these participants, who appear to completely lack awareness of the target’s location and who perform no better than chance in the generation test, nonetheless responded much faster to repeating than random patterns in the earlier contextual cuing part of the experiment.^4^

This pattern is therefore a clear replication of the post hoc selection method that has been used in many other contextual cuing experiments (e.g., Chun & Jiang, 2003). But is it evidence for true unconscious knowledge, or is it simply an inevitable consequence of regression to the mean? We can address this question in two different ways. First, we can ask whether the observed degree of regression to the mean in the selected participants’ mean priming score (*X*), given their mean awareness score (*Y*), is in line with Eq. 1. From the *X*–*Y* correlation (*r* = 0.10) and the awareness *z* score (-1.04), we can calculate the expected priming *z* score if it were purely a consequence of regression:

$$ z(X)=r\kern0.5em z(Y)=0.10\times -1.04=-0.10. $$

This value falls inside the 95 % confidence interval of the observed mean priming *z* score in the selected subgroup, *M* = 0.27, 95 % CI [-0.30, 0.83]. Thus, the observed level of priming in the unaware subgroup is consistent with regression to the mean being the only causal process.

There is a second and even more compelling way of testing the different predictions made by the two accounts, namely to compare the selected participants’ scores on Tests 1 and 2. Recall that the regression account predicts that extreme scores on Test 1 will regress to the mean on Test 2. In Fig. 6b, each participant is depicted by two data points, one (open circles) showing their scores on Test 1 and the other (red crosses) showing Test 2, with each point having the same performance score (*X* value). The green and blue diamonds show the subgroup’s mean scores for each test. It is clear that the regression account is strongly supported: Most of the participants (9/14) score better on Test 2 than Test 1, with only two showing the opposite pattern (there were three ties). This improvement is significant, *M* = .08, 95 % CI [0.02, 0.14], and provides no support for the assumption on which the post hoc selection method rests. The method construes the selected participants as being truly unaware and therefore predicts that their low report scores should be maintained from Test 1 to Test 2.

It is important to emphasize that regression is a nondirectional process. Thus, data selected on the basis of extreme low scores on Test 1 will regress toward the mean on Test 2, but by the same token extreme low scores on Test 2 will regress toward the mean on Test 1: The mean change for the latter is about the same size, *M* = 0.10, 95 % CI [0.04, 0.15]. Moreover, the same applies to high scores. The regression account predicts that participants with high awareness scores on Test 1 will have lower scores on Test 2. This, again, holds true: The mean awareness score of participants selected for high scores (≥0.35) on Test 1 drops by -0.08 scale units, 95 % CI [-0.01, -0.15], on Test 2.

There is nothing special about this example or about the relationship between performance and report. If the height of all men in England is measured and a quantile, such as the upper quartile, is formed, the estimate of their average height will be biased upward. Measuring height is as imperfect as measuring anything else. The upper quartile will contain a disproportionate number of men for whom the measurement error is positive, creating a bias in measuring their true heights. To be concrete, the average height of men in England is about 175 cm (*σ* = 10). If a small amount of measurement error is assumed (*M* = 0 cm, *σ* = 3), and height is normally distributed, it is found (by simulation) that the average height of the upper quartile is 188.3 cm. However the true mean height of these men is a centimeter lower, 187.2 cm.^5^

The reason for this (just as in the use of post hoc selection in studies of unconscious cognition) is that the measurement of height in this example is being used for two purposes: to construct the subsample and to estimate the mean in the subsample. If two independent height measures were taken for these two purposes, then regression would be allowed to take its natural course, and the estimate of height in the subsample would be unbiased. This leads to the obvious recommendation about future use of post hoc selection in research on unconscious cognition: It must be based on two independent measures of awareness. I amplify this recommendation later.

The reliability of the awareness measure in the data described above, calculated as the correlation across participants between their awareness scores on Tests 1 and 2, is a mere 0.37. This means that the measurement error on *Y*, *e*_*Y*_, is substantial. The reliability estimate is low, but it is important to highlight that other research has also obtained reliability estimates far below 1.0 for tests of reportable knowledge. For instance, Buchner and Wippich (2000) reported values between and 0.41 and 0.88 for explicit recognition and recall tests in memory experiments. The figures they obtained for priming tests were even lower: 0.13–0.44 (see also LeBel & Paunonen, 2011; Ward, Berry, & Shanks, 2013).

## The prevalence of post hoc data selection in research on unconscious cognition

In this section I briefly describe the extent to which the method described above has been employed in research on unconscious cognition. This review highlights the fact that post hoc selection has been in regular use for over a century to address a broad range of specific questions about unconscious processing. It would be impossible to systematically collect all such studies (in part because no consistent name for the method is used) and because it is applied in so many different contexts, but it is clear that dozens of studies have based their conclusions in whole or part on this analytic technique.

The first use was by Peirce and Jastrow (1884), in one of the most famous experiments from the early history of psychology. In tests of pressure discrimination, Peirce and Jastrow used an apparatus that allowed weights to induce different pressures on the finger. Two pressures were presented in succession and followed by a judgment about which was greater, and, finally, a confidence rating from 0–3, where 0 “denoted absence of any preference for one answer over its opposite, so that it seemed nonsensical to answer at all,” and 3 “denoted as strong a confidence as one would have about such sensations” (p. 77). Peirce and Jastrow found that very similar pressures could be reliably discriminated better than pure guessing, even on those occasions when a rating of zero was made. An early use in vision was an analogous experiment by Williams (1938), who presented one of three stimuli (a circle, triangle, or square) at near-threshold intensity for participants to identify at a distance of about 10 feet (~3 m). Each response was accompanied by one of three reports: that the figure was clearly seen, that something was seen, or that nothing was seen. Williams observed that identification was reliably better than chance, even for unseen stimuli.

It is instructive to consider these studies alongside those of Sidis (1898). Although the research of Peirce and Jastrow (1884) and Sidis (1898) is often discussed together, their methods were—for present purposes—different in a crucial respect. Sidis placed participants far enough away from a card that they could not consciously make out the letter or number printed on it. He reports that

“he saw nothing but a dim, blurred spot or dot. The subject had to name some character which that particular dot shown might possibly be. ‘It is nothing but mere guess,’ commented the subjects” Sidis (1898, p. 170).

Nevertheless, the characters were identified with accuracy much greater than would be expected by pure guessing. Although the empirical conclusion is the same, Sidis’s findings are not susceptible to a regression-to-the-mean artifact because no selection was required: By removing the card to a sufficient distance, Sidis ensured that *all* trials were unconscious ones, not just those chosen post hoc on the basis of the participant’s report.

In a famous and influential study, Lazarus and McCleary (1951) first paired nonwords with a shock unconditioned stimulus and subsequently observed reliable skin conductance responses when the nonwords were briefly presented, and even on trials (selected post hoc) where the participant was unable to report the stimulus. Many early examples are reviewed by Dixon (1971), in the case of subliminal perception, and Brewer (1974), in the case of Pavlovian and instrumental conditioning. Brewer notes the existence of at least 31 experiments published during the 1950s on just one particular topic, which divided aware and unaware participants to study verbal operant conditioning.

The studies described in Table 1, which include articles in highly prestigious journals such as *Science* and *Nature*, highlight the breadth of applications of the post hoc method. For example, selection has been applied to participants and to trials. It has been applied in primates (Supèr, Spekreijse, & Lamme, 2001) as well as humans, with “reports” made nonverbally. It has been employed in studies of social learning (Heerey & Velani, 2010), language (Paciorek & Williams, 2015), and emotion processing (Sweeny, Grabowecky, Suzuki, & Paller, 2009), as well as in the clinical domain (Mogg, Bradley, & Williams, 1995).

**Table 1:** Examples of studies which have employed post hoc data selection at the level either of participants or trials/items, and their major conclusions.

Study	Field	Data selection	Major finding
Clark and Squire (1998)	Pavlovian conditioning	Participants classified as unaware by postconditioning verbal reports	Unconscious (procedural) delay but not trace eye-blink conditioning
Schultz and Helmstetter (2010)	Pavlovian conditioning	Participants classified as unaware in a concurrent expectancy test	Unconscious autonomic conditioning
Jones, Fazio, and Olson (2009)	Pavlovian conditioning	Participants classified as unaware by postconditioning verbal reports	Unconscious evaluative conditioning has an attributional basis
Willingham, Nissen, and Bullemer (1989)	Sequence learning	Participants classified as unaware by postlearning verbal reports	Unconscious sequence learning
Sanchez, Gobel, and Reber (2010)	Sequence learning	Participants classified as unaware in postlearning recognition and recall tests	Unconscious perceptual-motor sequence learning
Weiermann and Meier (2012)	Sequence learning	Participants classified as unaware by postlearning verbal reports	Unconscious sequence learning in young adults, but not children or older adults
Batterink, Reber, Neville, and Paller (2015)	Statistical learning	Participants classified as unaware on a recognition test	Unaware participants show statistical learning
Harris, Schwarzkopf, Song, Bahrami, and Rees (2011)	Vision	Trials on which participants reported no awareness of visual stimulus	Brightness contrast for invisible stimuli
Mogg, Bradley, and Williams (1995)	Vision	Participants classified as unaware in a prime discrimination test	Subliminal threat stimuli prioritized by anxious but not depressed participants
Chun and Jiang (1998)	Visual search	Participants classified as unaware in postlearning recognition and verbal report tests	Unconscious contextual cuing of visual search
Geyer, Shi, and Müller (2010)	Visual search	Contexts or participants classified as unaware in a postlearning recognition test	Unconscious contextual cuing and contextual priming of visual search
Supèr et al. (2001)	Primate vision	Trials classified as “unseen” by saccadic eye movement report	Late but not early processing suppressed for unseen stimuli
Charles, King, and Dehaene (2014)	Error detection	Trials classified as unaware by subjective report	Visual stimuli and responses, but not accuracy, coded unconsciously
Sklar et al. (2012)	Arithmetic	Participants classified as unaware by postpriming forced-choice test	Unconscious arithmetic
Paciorek and Williams (2015)	Language	Participants classified as unaware by postlearning questionnaire	Unconscious semantic generalization
Muscarella, Brintazzoli, Gordts, Soetens, and Van den Bussche (2013)	Consumer behavior	Participants classified as unaware by postpriming forced-choice test	Unconscious priming from brand logos
Ryan, Althoff, Whitlow, and Cohen (2000)	Memory	Trials on which conscious report of relational manipulation failed	Eye movements reveal unconscious relational memory in normal adults, but not amnesic individuals
Hannula and Ranganath (2009)	Memory	Trials on which conscious recognition failed	Eye movements reveal unconscious relational memory driven by hippocampal activity
Stark and McClelland (2000)	Memory	Old and new items judged new in a recognition test	Unconscious repetition priming for unrecognized words and nonwords
Duke, Fiacconi, and Köhler (2014)	Memory	Participants classified as unaware in a prime discrimination test	Fluency and positive affect unconsciously influence familiarity, but not recollection
Slotnick and Schacter (2004)	False memory	Old and related items judged new in a recognition test	Unconscious neural signals distinguish true and false memories
Jensen, Kirsch, Odmalm, Kaptchuk, and Ingvar (2015)	Pain perception	Participants classified as unaware in a postconditioning recognition test	Unconscious conditioned analgesia/hyperalgesia
Rugg et al. (1998)	Cognitive neuroscience	Old and new items judged new in a recognition test	Neural activity for misses, greater than for correct rejections, reflects unconscious memory
Daselaar, Fleck, Prince, and Cabeza (2006)	Cognitive neuroscience	Old and new items judged new in a recognition test	Hippocampal activity for misses, equivalent to that for hits, reflects unconscious memory
Koivisto, Mäntylä, and Silvanto (2010)	Cognitive neuroscience	Trials classified as unaware by subjective report	Transcranial magnetic stimulation impairs unconscious motion detection
Heerey and Velani (2010)	Social cognition	Participants classified as unaware by postlearning forced-choice test	Unconscious learning of nonverbal social cues
Pessiglione et al. (2007)	Motivation	Participants classified as unaware by a forced-choice test	Unconscious motivation of physical effort
Sweeny, Grabowecky, Suzuki, and Paller (2009)	Emotion processing	Participants classified as unaware by postpriming forced-choice test	Unconscious affective priming can induce long-lasting biases

Trial-based selection has been applied not only when binary awareness measures have been made but also with finer categorizations: in the study by Koivisto, Mäntylä, and Silvanto (2010), for instance, awareness of motion was assessed by a 4-point scale where 1 = *I did not perceive any motion at all*, 2 = *I might have perceived motion, but I did not have any idea of its direction*, 3 = *I did not actually see the direction of the motion, but I may have been able to sense or guess its direction*, and 4 = *I saw the direction of the motion*. In this case, post hoc analysis was confined to trials where a rating of 1 was given.

In many subliminal perception experiments (such as those of Koivisto et al., 2010), selection at the level of trials depended on the individual report for that trial, and random fluctuations in attention presumably determined the report and hence whether the trial was selected. In other studies, selection has been done at the level of items rather than trials. For example, Geyer, Shi, and Müller (2010) used eight distinct contexts (patterns of distractors in a visual search task) and at the end of the experiment assessed participants’ awareness of each context by a recognition test. They then applied post hoc selection on a context-by-context basis to pool only those contexts for which awareness, across participants, was lacking.

This brief review provides an indication of the scale and breadth of usage of the post hoc selection method. One other noteworthy point is that none of these studies considered the possibility that regression to the mean could come into play as a result of using this analytic approach, despite the widespread warnings in other domains within psychological research (Campbell & Kenny, 1999). Of course, it is not being suggested that all of the conclusions of all studies employing the method are invalid. In many cases, the method contributes only a small part of the evidence on which authors drew their conclusions. But to the extent that conclusions do depend on the method, they should be regarded as unsound.

Stated differently, it must be the case that for each of the examples included in Table 1 there exists a model that makes no distinction between conscious and unconscious processes at the level of the latent processes, but that nonetheless can predict the key qualitative pattern simply as a result of regression to the mean. Above-baseline indirect performance in participants/items/trials classified as unconscious can arise simply for this reason, as the models described previously demonstrate. Of course, whether such models can explain the magnitude of the key effect in each particular case, and the complete response pattern observed, would have to be determined on a case-by-case basis (the next section provides one example). But unless it can be shown that the regression artifact is insufficient, the results reported in these (and many other) studies fall short of demonstrating unconscious mental processing.

## Subliminal reading and arithmetic

To this point, the conditions in which post hoc data selection will cause regression artifacts have been characterized, a prediction of the account has been tested and confirmed in the data of Smyth and Shanks (2008), and the scale and breadth of usage of the method in contemporary research on implicit cognition has been reviewed. In this final major section, the regression account is applied to a recent and high-profile case in order to highlight how post hoc data selection can lead researchers to draw unfounded conclusions.

Research using continuous flash suppression by Sklar et al. (2012) appears to show that reading and doing arithmetic can be achieved unconsciously. Sklar et al. reported nine experiments using continuous flash suppression (CFS), in which a stimulus presented to one eye can be rendered invisible by flashing mask patterns presented simultaneously to the other eye. CFS is an attractive technique compared to more traditional subliminal perception methods for studying unconscious processing because very brief and precise stimulus timings are not required. In these experiments Sklar et al. presented either linguistic statements or arithmetic expressions to the suppressed eye and measured the time that it took the stimuli to break suppression or their influence on related decisions. In only three of these experiments, however, was an objective awareness test employed, and hence I focus on one of these (Experiment 6; the following analysis also extends to the other two experiments). In this experiment the primes were arithmetic strings, such as “9 - 3 - 4 =” and the target was the correct (compatible: “2”) or an incorrect (incompatible: “3”) digit. Sklar et al. reasoned that if participants took longer to read the target aloud in the incompatible than in the compatible trials, this would be evidence of information being extracted from the prime. They obtained such a priming effect, though only with subtraction expressions.

**Fig. 7:**
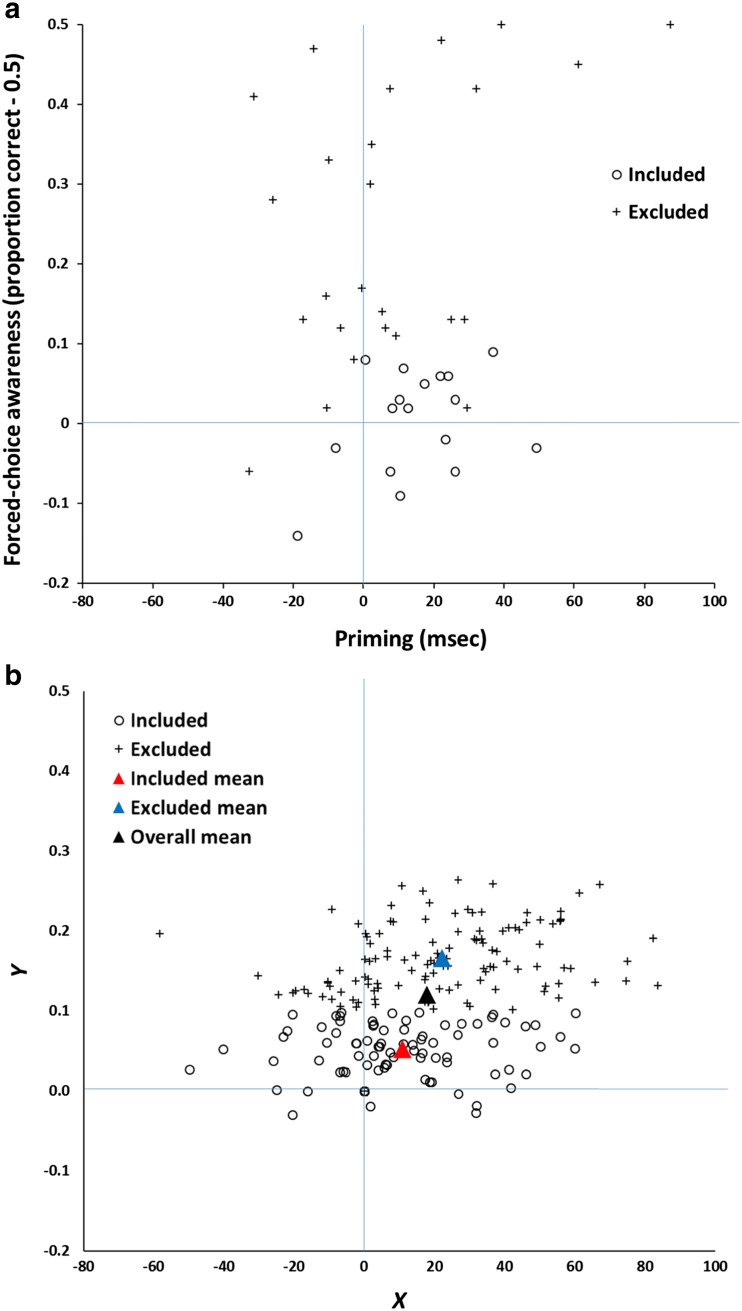
**a** Priming and forced-choice performance in Sklar et al.’s (2012) Experiment 6. Each point represents one participant (*n* = 42). The *x-*axis represents the facilitation (ms) for compatible compared to incompatible target stimuli, and the *y-*axis represents forced-choice accuracy (proportion correct – 0.5). Participants (*n* = 25) were excluded either if their awareness score was greater than chance by a binomial test (*n* = 21) or if they explicitly reported awareness of the primes (*n* = 4). **b** Simulation of the results shown in Panel A. Data (*n* = 200) were generated according to Eqs. 8 and 9. Open circles represent simulated participants included by the post hoc method on the basis of *Y* scores less than or equal to 0.1. Crosses are nonselected participants. Triangle symbols represent the mean scores of the entire sample (black), the included (red) subsample, and the excluded (blue) subsample. The mean *X* score (priming) is greater than zero in all samples, including those scoring below chance on the awareness measure. (Color figure online)

In the subsequent objective awareness test, participants were presented with the same prime stimuli, but now were required to explicitly report whether the first digit in the expression was odd or even. Performing at chance (50 %) in this forced-choice test would be evidence that no information about the expression was consciously detected. Putting aside issues such as task difficulty (Pratte & Rouder, 2009) and the reliance on null hypothesis significance testing (Vadillo et al., 2016) in the awareness check, I interpret it (as Sklar et al., 2012, did) as a valid measure of prime awareness. The results are shown in Fig. 7a (in line with Sklar et al.’s, 2012, analysis, 0.50 has been subtracted from the proportion correct so that chance is now zero). The first and most notable feature of these is the overwhelming tendency for participants to score above chance in the awareness test (*M* = 0.15, 95 % CI [0.09, 0.21], *d* = 0.81). Indeed, 34/42 participants did so and hence the majority of Sklar et al.’s participants were aware of the primes. The priming effect, in contrast, is both small (*M* = 10.8 ms, 95 % CI [3.3, 18.3], *d* = 0.45) and less consistent, with only 29/42 participants showing a numerical effect.

**Figure 8:**
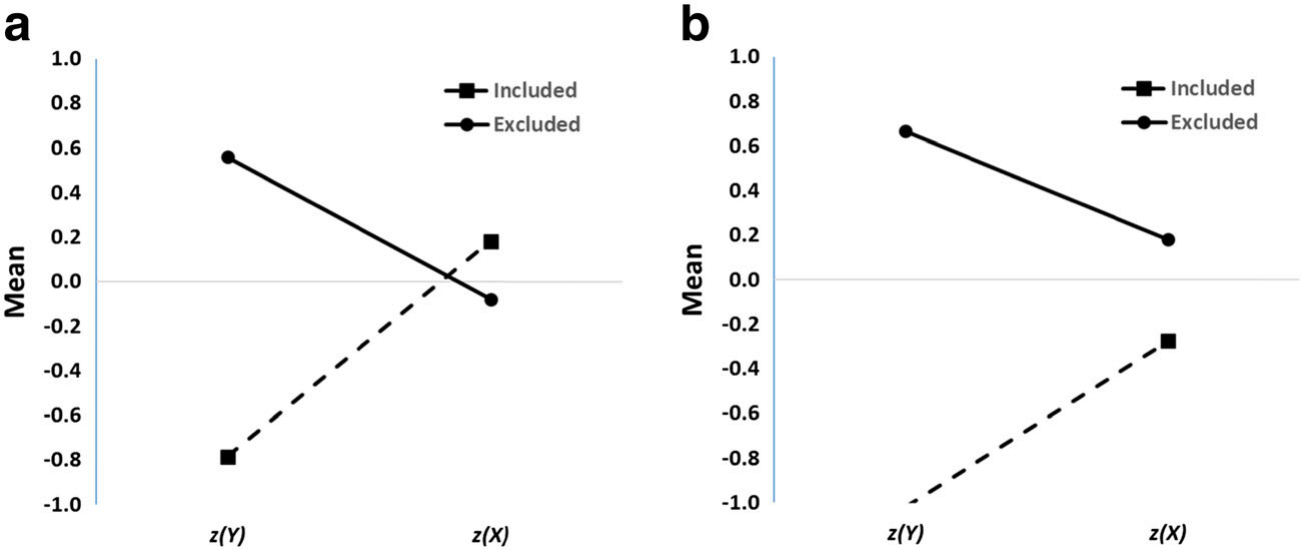
Galton squeeze diagrams for the data in Fig. 7a (left panel) and b (right panel). Each diagram shows the mean *z* scores of the *Y* (awareness) measure for the included and excluded participants plotted against the equivalent *z* scores for *X* (priming). The funnel pattern is regression to the mean.

On what basis could Sklar et al. (2012) conclude that unconscious arithmetic calculations were taking place in this experiment? They did so as a result of employing a post hoc data selection analysis, eliminating participants who performed above chance on the awareness test. Specifically, participants (*n* = 25) were excluded either if their awareness score was greater than chance by a binomial test (*n* = 21) or if they explicitly reported awareness of the primes (*n* = 4). Thus, the included participants in Fig. 7a scored significantly above chance for priming (and +0.181 *z* scores from the mean priming score), but not for awareness (-0.785 *z* scores from the mean score). This, of course, is regression to the mean, illustrated in Fig. 8a via a Galton squeeze diagram based on *z*-transformed scores. From Eq. 1 and the *X*–*Y* correlation (*r* = 0.198), we can calculate the expected consequence of regression as 0.198 × -0.785 = -0.155, which is the expected priming *z* score. This score falls (just) inside the 95 % CI of the observed score, [-0.163, 0.525]. Thus the observed level of priming in the “unaware” subgroup is not sufficiently greater than the level predicted purely by regression to the mean to rule out the latter as the sole process in operation.^6^

Consistent with regression, the excluded (aware) participants show the exact converse pattern, a mean awareness score well above the group mean and a mean priming score much closer to the overall mean. When both measures (awareness and priming) are above chance in the entire sample, the selection procedure creates conditions in which the subsample who meet the cutoff are more likely to score significantly above chance in priming than they are in awareness. Sklar et al.’s results are therefore not a scientific discovery, but a statistical inevitability.

Two other points are worth noting. First, if we simply focused on the eight participants who scored at or below chance on the awareness test (all the data points in Fig. 7a below the *x-*axis, *p* > .05), reliable evidence of priming would not be seen. Second, Sklar et al.’s exclusion criterion was skewed in that it led to seven participants being included who scored below chance for awareness, but 10 who scored above chance. Luckily for Sklar et al., all 10 of the latter had nonnegative priming scores. Ironically, the skew is even greater in Sklar et al.’s Experiment 7, where of 30 participants included in the analysis, only 7 scored below chance for awareness and 20 scored above. This is a significant skew (*p* < .01), indicating that at least on this categorical measure, the sample supposedly showing unconscious priming in fact performed slightly but significantly above chance on the awareness test.

In sum, these results would arise even if prime awareness were a necessary condition for priming. This point is now reinforced via a simulation study of the key pattern Sklar et al. (2012) obtained. I present a simple model of their data based on two key components: (a) in contrast to Sklar et al.’s conclusion, it assumes that priming and awareness depend on a common underlying representation rather than on distinct unconscious and conscious ones, and (b) it permits regression to the mean to play its inevitable role.

### Simulating priming and awareness in Sklar et al.’s Experiment 6

Each participant is assumed to form some internal representation *S* of the suppressed stimuli presented in the experiment. This representation is normally distributed with mean 1.0 and standard deviation 0.5. This representation causes a priming effect *X* for the participant, but the magnitude of this effect depends on a noisy process:

8 $$ X={\mu}_X\kern0.5em S+{e}_X. $$

In this equation, *μ*_*X*_ is a free parameter that scales the internal representation *S*, and *e*_*X*_ is random noise added to the process. The latter is assumed to have a mean of zero and standard deviation of 24, while *μ*_*X*_ is set to a value of 18.

Scores on the forced-choice awareness (*Y*) test are modeled in a similar way. The very same value of *S* for that stimulus is taken, but added with an independent source of noise:

9 $$ Y={\mu}_Y\kern0.5em S+{e}_Y. $$

Here, *μ*_*Y*_ scales *S* appropriately onto the awareness scale and *e*_*Y*_ is a further source of noise, independent from *e*_*X*_. It is assumed that *e*_*Y*_ has a mean of zero and a standard deviation of 0.04, while *μ*_*Y*_ is set at 0.12. The precise values of these parameters are not crucial. They simply ensure that *X* and *Y* have means and standard deviations fairly similar to those observed in the experiment.

The critical point to note is that in this model, the observed amount of priming and the accuracy of forced-choice identification for a given participant depend on a common latent variable for that participant, *S*. The only difference is that this variable is scaled differently in the two tasks and combined with independent sources of noise. The model therefore assumes that there is no fundamental difference between what the two tests are measuring: It makes no assumption that the priming test measures some form of unconscious knowledge that cannot be accessed by the awareness test. Indeed, the model could not depart further from Sklar et al.’s explanation of their data, as (like the earlier models) it cannot predict priming in the true absence of awareness. To see this, note that mean awareness $\overline{Y}$ in Eq. 9 can only be at chance (zero) when mean $\overline{S}$ = 0. Since *S* is identical in Eqs. 8 and 9, then if $\overline{S}$ = 0, $\overline{X}$ = 0 as well and priming is predicted to be absent.

To simulate the experiment, 200 data points (participants) were generated from the model, each comprising a priming and an awareness measure. These were then segregated into those for which awareness was at or below the cutoff (0.1, based on the binomial test) and those for which it was above. The results are plotted in Fig. 7b. Because of the parameter settings, the generated data have similar means and standard deviations on each measure to those observed in the experiment. More important is that the mean priming for the simulated participants whose awareness fell at or below the cutoff was 10.9 ms, 95 % CI [5.8, 15.9], substantially above zero and within the estimate of the priming level Sklar et al. observed in their Experiment 6, *M* = 15.2, 95 % CI [6.9, 23.4]. Figure 8b shows that the degree of regression in the simulation is very similar to that observed in the experimental data.

Although the key finding (reliable priming in participants classified as unaware) arises across a large portion of the parameter space, the particular parameter values chosen for the simulation allow an additional constraint to be met. The awareness measure Sklar et al. employed has relatively high reliability, in the range 0.7–0.9 across Experiments 6 and 7 (A. Sklar, A. Goldstein, & R. Hassin, personal communication, February 9, 2016). The simulated awareness measure has comparable reliability (*r* = 0.76), calculated by generating two awareness scores *Y* with independent values of *e*_*Y*_ for each of 200 values of *S* and correlating the resulting *Y*’s. Thus, the key finding does not depend on especially low reliability in the awareness measure.

It is important to emphasize that the point is not to fit Sklar et al.’s data precisely, which would in any case be of little value given the small sample size of the experiment and ensuing high variability. Rather, the point is to highlight that regression to the mean must be a factor and is indeed sufficient to yield Sklar et al.’s key qualitative result, namely, above-chance priming in participants selected post hoc as being unaware. Because the model in Eqs. 8 and 9 is incapable of generating priming in the true absence of awareness, the pattern generated in Fig. 7b—and by inference the qualitatively identical one in Fig. 7a—must be due to something else. That something else is regression to the mean.

The regression account can be thought of as a “null” model, which assumes that awareness and performance depend on a common source. In focusing on this null model, it is important to avoid inferring that all data that are consistent with regression to the mean are uniquely supportive of this theoretical position. Put differently, finding that performance in unaware participants is approximately in line with what one would expect from regression to the mean does not automatically support the regression model over an alternative unconscious processing hypothesis. As always, data are only diagnostic between competing models when they permit precise measurement, which in turn requires adequate sample sizes and careful experimental design.

In sum, the model predicts a priming effect in participants whose awareness is near chance. It does this despite the fact that it embodies no distinction between conscious and unconscious processing. Crucial to the explanation is that the model’s predictions incorporate regression to the mean. Data selected on one dimension show regression in their scores on the other dimension.

## Relative ordering of means

A final observation about the regression analysis concerns its predictions for the relative mean performance scores of aware and unaware samples, versus the group mean. If *z(Y*|*Y* ≤ 0*)* and *z(Y*|*Y* > 0*)* are the *z*-transformed awareness measures of unaware and aware subgroups, and *z(Y)* is the group mean, then we know that these are ordered *z(Y*|*Y* > 0*)* > *z(Y)* > *z(Y*|*Y* ≤ 0*)*. That is, average awareness in the aware subgroup is greater than the overall mean, while that in the unaware subgroup is lower. From Eq. 1 it follows that *z(X*|*Y* > 0*)* > *z(X)* > *z(X*|*Y* ≤ 0*)* if *r* > 0. Performance should be greatest in the aware subgroup and lowest for the unaware subgroup, with the overall group mean falling in between. This pattern is evident in Fig. 7b, where performance is greatest for the aware (included) participants and lowest for the unaware (excluded) ones. The predicted ordering is reversed for negative *r*.

Some of the empirical results analyzed in this article are clearly at variance with this predicted ordering. For example, in the reanalysis of the data from Smyth and Shanks (2008, Experiment 1), contextual cuing was greater in the unaware participants than the overall mean (116 vs. 81 ms). Likewise, in Sklar et al.’s (2012) Experiment 6, mean priming was 15 ms in unaware participants, compared to an overall group mean of 11 ms. In neither of these cases is the difference statistically significant, but they do, nonetheless, violate the predicted ordering and must, if regression is the only process operating, be attributed to sampling error. Future studies, employing sufficiently large samples to permit high-powered tests of the relative ordering of performance scores in aware and unaware subgroups, will be of considerable interest.

## Conclusions

It has been appreciated for many years that regression to the mean must be carefully taken into account when evaluating interventions in clinical and educational psychology and elsewhere (see Campbell & Kenny, 1999; Fiedler & Unkelbach, 2014). This article demonstrates that an entirely different field within psychology, concerned with characterizing unconscious mental processes, regularly uses an analytic technique in which regression to the mean inevitably complicates the inferences that can be drawn. Indeed once regression is considered, the results of many of these studies may turn out not to require postulating distinct unconscious processes at all.

What recommendations can be made about future use of post hoc selection in research on unconscious cognition? This analysis points to an obvious suggestion: If it is to be used at all, it should ideally be based on two independent measures of awareness. If one measure is used to select the “unaware” sample and the second to provide an independent estimate of awareness in this sample, regression to the mean will tend to eliminate bias in the second measure. This recommendation should not be onerous to implement. It does not require two completely different awareness tests; the odd and even trials from a single test, for example, would be adequate. What one therefore asks is whether a subgroup created by applying a cutoff on Test 1 (a) shows a significant performance effect and (b) continues to score at or below chance on Test 2.

Use of two independent awareness measures should be the “gold standard”. But if only one awareness measure is available, then a simple test, as used above in the reanalyses of the Smyth and Shanks (2008) and Sklar et al. (2012) datasets, is to compare the observed performance measure in the “unaware” sample against the score that would be predicted by regression to the mean alone (i.e., Eq. 1). Thus, one can estimate the magnitude of a performance effect *X* if it arose purely from regression to the mean and compare this to the observed magnitude. If the latter is substantially larger than the former, then a true performance effect can be inferred. Of course, a high-powered test is required if this comparison is to be diagnostic: In the analyses reported above, the confidence intervals on the performance scores were very wide. Other methods are also available but are less satisfactory because they rely on assumptions that may be difficult to validate. For instance, methods for statistically correcting for regression to the mean have been developed (see Hsu, 1995, for discussion).

Better still is to avoid use of post hoc selection entirely. One approach is to employ experimental manipulations to ensure that the entire group is unaware, or on a participant-by-participant basis. For example, in an implicit learning experiment, the length of the training phase could be titrated to ensure that group-level awareness is not significantly greater than chance. Under such circumstances no post hoc selection is required. Of course, care must be taken to ensure that scores on the awareness test really are very close to chance: A large sample is likely to be required to ensure narrow confidence intervals, or a clear Bayes factor favoring the null, on the awareness measure (Dienes, 2015; Vadillo et al., 2016).

With this in mind, it is clear how Sklar et al. (2012) could obtain stronger evidence for unconscious arithmetic. In their experiments, the prime stimulus was presented in continuous flash suppression for 1,700 or 2,000 ms and, as noted previously, participants performed as a group significantly better than chance in the forced-choice awareness test. Thus, one strategy would be to reduce the prime presentation time for all participants to try to find an exposure duration at which a group-level priming effect could still be detected, but with chance-level awareness. Under such conditions, no post hoc selection would be required.

It is interesting to note that, at least in the field of subliminal perception, participant-by-participant stimulus calibration was once the norm (see Holender, 1986). Researchers routinely employed staircase methods in which the duration of a prime stimulus was increased and decreased in order for each participant’s awareness threshold to be determined. Then, in the main subliminal perception task, the stimulus was presented below each participant’s threshold. Under these circumstances, no post hoc selection is required, and any group-level priming can legitimately be taken as evidence for unconscious processing. Gradually, however, this practice dwindled, perhaps because it is time-consuming to determine each participant’s individual threshold, and was replaced by group-level subjective and objective tests and, in some cases, post hoc data selection. Thus, another way in which researchers such as Sklar et al. (2012) could obtain stronger evidence is to determine each participant’s awareness threshold individually, and ask whether priming occurs when stimuli are presented below threshold.

Preacher et al. (2005) cautioned “that the conclusions that can be based on the results of EGA [extreme groups analysis], although sometimes useful, are limited relative to those based on analysis of full-range, continuous data” (p. 190). Given the concerns identified in this article, it is reasonable to ask whether traditional regression techniques applied to the entire *X*–*Y* dataset might answer the key questions researchers are interested in concerning unconscious processing, without all the problems associated with regression to the mean. Rather than excluding data, analytic techniques can be employed that retain all data and adopt different logic to estimate unconscious processing.

An example is the development by Greenwald et al. (1995) of a regression method that tests for the value of the *X* intercept when *Y* = 0. If this intercept is positive, then it seems reasonable to infer that performance must be driven at least in part by unconscious information. Greenwald and colleagues found evidence of subliminal perception by this method in a large study including 1,431 participants. Unfortunately even this technique is not immune to problems of measurement error and regression to the mean. Miller (2000; see also Dosher, 1998; Sand & Nilsson, 2016) conducted a series of simulations and concluded that measurement error systematically biases the estimation of the intercept upward, such that it appears positive when it should truly be zero. The present critical appraisal of the post hoc method parallels Miller’s (2000) equivalent appraisal of the regression method.

Alternatively, instead of focusing solely on examining the relationship between manifest variables assumed to accurately measure different forms (conscious and unconscious) of mental processing, a much more powerful approach is to measure the relationships between the latent variables assumed to underlie these manifest variables, using well-established techniques such as structural equation modeling or confirmatory factor analysis. Some examples of this approach show that it has considerable promise (Kaufman et al., 2010; Rünger, Nagy, & Frensch, 2009; Salthouse, McGuthry, & Hambrick, 1999; Woltz & Shute, 1993).

## Footnotes

1 Employing a statistical test applied participant by participant introduces a striking bias that can lead to spurious evidence for an unconscious effect. Suppose that a subgroup of apparently unaware participants is selected post hoc and is found to have a mean performance score significantly greater than zero. Even though each individual participant’s awareness score might not be significantly greater than chance, it is the mean awareness score from the entire subgroup that matters, and this may well be significant. For example, Clark and Squire (1998) adopted such an approach (see Lovibond & Shanks, 2002).

2 Note that statisticians (see Krause, 2009; Samuels, 1991) distinguish between “reversion” and “regression” to the mean, and indeed Samuels (1991) proposes that the term *reversion* to the mean should be used to describe the property captured in Fig. 1a. I continue to use *regression* here because it is a term more familiar to behavioural researchers, and Samuels’ proposed usage is not universally accepted.

3 In the linear regression model *Y* = *a* + *bX*, the slope *b* is given by *b* = *r σ*_Y_/*σ*_X_. If the variables are standardized so that *σ*_Y_ = *σ*_X_ and the intercept *a* = 0, it follows that *z(Y)* = *r z(X)*. See Cohen et al. (2003, Ch. 2).

4 Smyth and Shanks (2008) reported results of various post hoc selection analyses across both participants and trials. Lest the reader conclude that we were guilty of the analytic fallacy being highlighted in this article, it should be pointed out that those analyses were only included at the request of a reviewer.

5 Note that the latter is not an accurate estimate of the height of men in the upper quartile of the population, but it is the average height of those men whose *measured* height (including error) places them in the upper quartile. This group will erroneously include some whose true height should not put them in the quartile and exclude some men who should be in this quartile but whose true height is underestimated as a result of measurement error. The correct estimate of the true height of the upper quartile is 187.7 cm.

6 Sklar et al.’s (2012) Experiment 7 was similar to Experiment 6. Priming in participants selected post hoc as unaware (*N* = 30) was 13.3 ms, 95 % CI [1.8, 24.7]. This equates to 0.26 *z* scores, 95 % CI [-0.11, 0.63], above the group mean priming score, while the subgroup’s awareness was -0.78 *z* scores below the mean score. From Eq. 1 and the *X*–*Y* correlation (*r* = -0.18), the expected priming *z* score as a consequence of regression alone is -0.18 × -0.78 = 0.14, which falls inside the 95 % CI of the observed score. Thus, the results of both experiments are in line with the regression account.
